# Depression-Associated *Negr1* Gene-Deficiency Induces Alterations in the Monoaminergic Neurotransmission Enhancing Time-Dependent Sensitization to Amphetamine in Male Mice

**DOI:** 10.3390/brainsci12121696

**Published:** 2022-12-10

**Authors:** Maria Kaare, Mohan Jayaram, Toomas Jagomäe, Katyayani Singh, Kalle Kilk, Kaie Mikheim, Marko Leevik, Este Leidmaa, Jane Varul, Helis Nõmm, Kristi Rähn, Tanel Visnapuu, Mario Plaas, Kersti Lilleväli, Michael K. E. Schäfer, Mari-Anne Philips, Eero Vasar

**Affiliations:** 1 Institute of Biomedicine and Translational Medicine, Department of Physiology, University of Tartu, 19 Ravila Street, 50411 Tartu, Estonia; 2 Centre of Excellence in Genomics and Translational Medicine, University of Tartu, 50411 Tartu, Estonia; 3 Institute of Biomedicine and Translational Medicine, Laboratory Animal Centre, University of Tartu, 14B Ravila Street, 50411 Tartu, Estonia; 4 Institute of Biomedicine and Translational Medicine, Department of Biochemistry, University of Tartu, 19 Ravila Street, 50411 Tartu, Estonia; 5 Institute of Molecular Psychiatry, Medical Faculty, University of Bonn, 53129 Bonn, Germany; 6 Department of Anesthesiology, Focus Program Translational Neurosciences, Research Center for Immunotherapy, University Medical Center of the Johannes Gutenberg-University, 55131 Mainz, Germany; 7 Focus Program Translational Neurosciences, Johannes Gutenberg University Mainz, 55131 Mainz, Germany; 8 Research Center for Immunotherapy, Johannes Gutenberg University Mainz, 55131 Mainz, Germany

**Keywords:** Negr1, depression, dopamine, serotonin, genetic models

## Abstract

In GWAS studies, the neural adhesion molecule encoding the neuronal growth regulator 1 (*NEGR1*) gene has been consistently linked with both depression and obesity. Although the linkage between *NEGR1* and depression is the strongest, evidence also suggests the involvement of *NEGR1* in a wide spectrum of psychiatric conditions. Here we show the expression of NEGR1 both in tyrosine- and tryptophan hydroxylase-positive cells. *Negr1*^−/−^ mice show a time-dependent increase in behavioral sensitization to amphetamine associated with increased dopamine release in both the dorsal and ventral striatum. Upregulation of transcripts encoding dopamine and serotonin transporters and higher levels of several monoamines and their metabolites was evident in distinct brain areas of *Negr1*^−/−^ mice. Chronic (23 days) escitalopram-induced reduction of serotonin and dopamine turnover is enhanced in *Negr1*^−/−^ mice, and escitalopram rescued reduced weight of hippocampi in *Negr1*^−/−^ mice. The current study is the first to show alterations in the brain monoaminergic systems in *Negr1*-deficient mice, suggesting that monoaminergic neural circuits contribute to both depressive and obesity-related phenotypes linked to the human *NEGR1* gene.

## 1. Introduction

The 1p31.1 locus in the human genome, encoding the neuronal growth regulator 1 (*NEGR1)* gene, has been recently identified as one of the most significant risk loci for both depression [1,2,3,4,5] and obesity [6,7,8]. Transcriptome and protein analysis suggest increased expression of NEGR1 in depression patients; an increased level of *NEGR1* has been reported in the brain, namely in the dorsolateral prefrontal cortex (DLPFC) and in the hypothalamic area [5] of patients with major depressive disorder (MDD) in comparison with healthy controls. Additional data from MDD patients suggest that the functional impact of NEGR1 might involve systemic regulation, as significant upregulation of NEGR1 has been shown in the cerebrospinal fluid [9] and peripheral blood of MDD patients [10,11].

In the major studies that have recently linked NEGR1 with depression, the diagnosis has been specified as MDD (major depressive disorder) [1,2,5], broad depression [3], and unipolar depression [4]. Although the linkage between NEGR1 and depression is the strongest, evidence also suggests the involvement of NEGR1 in a wide spectrum of psychiatric conditions [12]. The levels of NEGR1 protein and transcripts are elevated in the post-mortem prefrontal cortex (PFC) [13] and DLPFC [14] of schizophrenic patients. NEGR1 has also been shown to be associated with intelligence [15], dyslexia [16], and autism spectrum disorders (ASD) [17,18]. In addition, a microdeletion in the *NEGR1* gene was described in two siblings who presented cognitive disabilities, attention deficit hyperactivity disorder (ADHD), speech problems, and one of them also had features of autism [19].

NEGR1 is a member of the IgLON superfamily of cell adhesion molecules (CAMs), which also include limbic system-associated membrane protein (Lsamp), neurotrimin (Ntm), and opioid-binding protein/cell adhesion molecules such as (Opcml) and IgLON5 [20]. IgLONs dimerize homophilically and heterophilically to shape synaptic connections and neural circuits by spanning cellular junctions (in trans) and/or at the same side of a junction (in cis) [20]. NEGR1 has been shown to interact with other IgLONs, such as NTM, through the first Ig domain [21]. Direct interaction between another IgLON, LSAMP, and NEGR1 has been shown in protein microarray experiments [22] and also confirmed in the mouse brain [23]. IgLONs act synergistically, each forming the context for the work of the other in the regulation of neural circuit formation, which manifests both at the level of neuronal morphology and behavior [20,24]. Therefore, NEGR1, together with other IgLONs, plays an important role in cell-to-cell adhesion and neurite outgrowth and synaptogenesis [25,26,27]. NEGR1 is reported to localize to the postsynaptic sites of dendritic and somatic synapses and is highly expressed in the cerebral cortex, hippocampus, and cerebellum during postnatal development [28].

Homozygous deletion of the *Negr1* gene in mice (*Negr1*^−/−^) induces no robust changes in sensory and motor development but causes impairment in social behavior and reversal learning deficits compared to WT littermates [27,29]. *Negr1*^−/−^ mice also displayed neuroanatomical alterations, such as enlargement of ventricles and a decrease in the volume of the whole brain, including the corpus callosum, hippocampus, and globus-pallidus. In addition, a decreased number of parvalbumin-positive inhibitory interneurons was evident in *Negr1*^−/−^ hippocampi [27]. In independently created *Negr1*^−/−^ mice, it has been shown that *Negr1* deficiency results in alterations in adult neurogenesis and hippocampal dentate gyrus (DG) synaptic transmission and leads to anxiety- and depression-like behaviors [30]. Szczurkowska et al. showed that the downregulation of *Negr1* in mice impairs neuronal migration and proper development of the somatosensory cortex resulting in behavioral phenotypes related to ASD [31]. We have also shown that *Negr1*^−/−^ mice eat smaller amounts of food both in case of standard and high-fat diets, which may be related to alterations in their metabolic profiles [32], but it is not clear if these mice have alterations in the motivational/reward system which may also explain reduced food intake. Again, the data from mice supports the strong associations of the *NEGR1* gene with both psychiatric and obesity-related phenotypes in human studies.

The monoamine hypothesis of depression states that dysfunction in the monoamine neurotransmitter system is the cause of the symptoms of depression. Although accumulating evidence also suggests the involvement of other pathways, monoamines still play a crucial role in mood disorders and are the main targets of antidepressant drugs that are currently available [33]. The expression of *Negr1* has been shown in both dopaminergic and serotonergic nuclei and pathways. *Negr1* is expressed in the whole fasciculus retroflexus, which serves as a molecular scaffold for dopaminergic axons that grow from the midbrain toward the habenula. Furthermore, the interaction partner of NEGR1, LSAMP, has been shown to mediate the guidance of the dopaminergic axons to the habenula [34]. High *Negr1* expression has been detected specifically in the islands of Calleja [27] which receive dense dopaminergic projections from the ventral tegmental area (VTA) and the substantia nigra [35]. The modest signal of *Negr1* and *Lsamp* was detected in the serotonergic neurons in the dorsal raphe of macaques. In another study, *Negr1* was identified as a differentially expressed gene across molecularly defined serotonergic neuron subtypes; the expression of *Negr1* was highest in the medial raphe, especially the ventral areas of the medial raphe (clusters R2 and R3), and was lowest in the dorsal raphe [36].

The expression of *Negr1* has been shown to be altered after the administration of several antidepressants that target monoaminergic neurotransmission. Chronic treatment with one of the most commonly used antidepressants, venlafaxine, a serotonin–norepinephrine reuptake inhibitor, has been shown to increase *Negr1* expression in the cerebral cortex in rats [37]. Carboni et al., however, showed a decrease of *Negr1* transcripts after the administration of selective serotonin reuptake inhibitors escitalopram in the hypothalamus and fluoxetine in the hippocampus in rodent models [38]. Moreover, tricyclic antidepressant nortriptyline downregulates *Negr1* in hippocampal primary neurons. Another study showed increased NEGR1 levels in human cell lines, which are treated with clozapine, which binds to both dopaminergic and serotonergic receptors, suggesting NEGR1 is a target of antipsychotic drugs [39].

Alterations in serotonergic neurotransmission, namely increased serotonin turnover, has been repeatedly described in *Lsamp*-deficient mice [40,41], which might be the explanation for the decreased anxiety and social deviations in these mice [42,43]. Decreased sensitivity to the stimulating locomotor effect of amphetamine has been described in mice deficient for *Lsamp* [39] and also in mice deficient for *Ntm* [44], further supporting the hypothesis of altered reactivity in the monoaminergic, especially dopaminergic neurotransmission, in mice deficient for IgLONs. Monoamines are also important regulators of reward and motivational processes. As mentioned above, feeding behavior is altered in *Negr1*-deficient mice. Characterization of monoaminergic neurotransmission in *Negr1*-deficient mice could clarify the mechanisms behind this observation.

The aim of this study was to assess the effects of deletion of *Negr1* on the monoaminergic circuitry as one of the mechanisms through which NEGR1 could be involved in the pathogenesis of depression and possibly in the pathogenesis of obesity.

## 2. Materials and Methods

### 2.1. Animals

Male wild-type (WT) mice and their homozygous *Negr1*-deficient littermates (*Negr1*^−/−^), generated and described previously in Lee et al. (2012) were used in the F2 background ((129S5/SvEvBrd × C57BL/6N) × (129S5/SvEvBrd × C57BL/6N)) in the present study [45]. Mice were group-housed in standard laboratory cages measuring 42.5 (L) × 26.6 (W) × 15.5 (H) cm, 10 animals per cage in the animal colony at 22 ± 1 °C, under a 12:12 h light/dark cycle (lights off at 19:00 h). A 2 cm layer of aspen bedding (Tapvei, Paekna, Estonia) and 0.5 L of aspen nesting material (Tapvei) were used in each cage and changed every week. Water and food pellets (R70, Lactamin AB, Kimstad, Sweden) were available ad libitum. Breeding and the maintenance of the mice were performed at the animal facility of the Institute of Biomedicine and Translational Medicine, University of Tartu, Estonia. All behavioral experiments were conducted between 8 a.m. and 5 p.m. Prior to experiment. each cohort of mice was in group housing conditions. During the experiment, cohort I mice went in single cages 6 days before the experiment and stayed there for the rest of the experiment. Mice were single-caged before the experiment since chronic administration of amphetamine increases fighting in mice [46]. The rest of the cohorts were in group housing conditions throughout all the experiments. The use of mice was conducted in accordance with the regulations and guidelines approved by the Laboratory Animal Center at the Institute of Biomedicine and Translational Medicine, University of Tartu, Estonia. All animal procedures were conducted in accordance with the European Communities Directive (2010/63/EU) with a permit (No. 150, 27 September 2019) from the Estonian National Board of Animal Experiments. Amphetamine and escitalopram were used as pharmacological agents to challenge monoaminergic neurotransmission in *Negr1*-deficient mice. Behavioral profiles were assessed together with monoamine and gene expression levels of related enzymes.

### 2.2. Acute and Chronic Amphetamine Treatment

**Figure 1:**
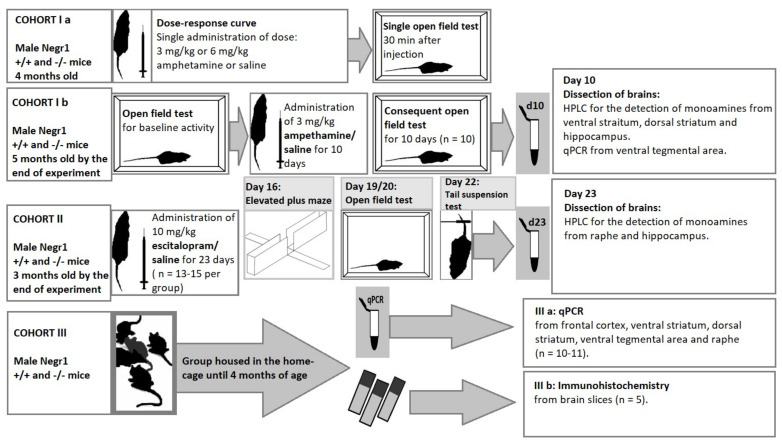
Schematic overview of the cohorts of mice and tests/measurements performed in the current study. For the estimation of the treatment of acute and chronic amphetamine (cohort I), two subgroups of mice were used: cohort Ia for the estimation of the dose curve (data shown in Supplementary Figure S1) and cohort Ib for the chronic amphetamine administration. An open field test for baseline activity of cohort Ib mice was performed 7 days before the administration of amphetamine. Cohort II was used for the estimation of the treatment of chronic escitalopram and cohort III was used for baseline measurement of gene expression and IHC stainings.

For the estimation of the treatment of acute and chronic amphetamine (cohort I), two subgroups of 5 months old mice were used: cohort Ia for the estimation of the dose–response curve and cohort Ib for the chronic amphetamine administration. A schematic overview of the cohorts of mice and tests/measurements performed can be found in Figure 1. All *Negr1*-deficient mice and their age-matched WT littermates were randomly assigned to groups (n = 10 per group). In the dose-response (acute administration) group (Ia), mice received a single dose of d-amphetamine in two different dosages: 3 mg/kg and 6 mg/kg or saline (Supplementary Figure S1). In the chronic amphetamine experiment (cohort Ib), all mice were tested in the open field test (I testing) two weeks prior to the amphetamine injections in order to detect their baseline motor/exploratory activity. Thereafter, cohort Ib mice received an i.p. injection of saline or 3 mg/kg amphetamine for 10 days, followed by a daily open field test 30 min after injection. The 3 mg/kg dose of amphetamine for repeated injection-induced behavioral sensitization has been shown to be sufficient in our earlier studies [47,48], still, the results were analyzed daily to make sure that there is no need to make corrections (decrease or increase) in the dose. Amphetamine (Sigma-Aldrich, St. Louis, MO, USA) was freshly prepared in a sterile, pyrogen-free, 0.9% solution of sodium chloride (B. Braun, Melsungen, Germany). In this experiment, we used the D-isomer of amphetamine (d-amphetamine) because l-amphetamine is a weaker agonist of the dopamine system. All drugs were injected intraperitoneally (i.p.) at a volume of 10 mL/kg 30 min before testing.

### 2.3. Escitalopram Treatment

All *Negr1*^−/−^ mice were age-matched with littermates and were tested at 2–3 months of age. *Negr1*^−/−^ and WT mice were randomly divided into groups that received an i.p. injection of either saline (B. Braun) or 10 mg/kg of selective serotonin reuptake inhibitor escitalopram (Sigma-Aldrich, Burlington, MA, USA) at a volume of 10 mL/kg for 23 consecutive days. The dosage of escitalopram was chosen based on Bregin et al. [41]. Mice were injected every day at 9 a.m. and allocated as follows: 13 mice in *Negr1*^−/−^ and 15 mice in WT escitalopram groups; in the saline groups, there were 13 mice in both WT and *Negr1*^−/−^ groups. Escitalopram (Sigma-Aldrich) was freshly prepared in a sterile pyrogen-free 0.9% solution of sodium chloride (B. Braun). Body weight was measured weekly for 8 weeks before administration of escitalopram and on days 1, 3, 5, 7, 9, 11, 13, 15, 17, 19, and 21 during the period of injections. Behavioral changes were evaluated in the elevated plus maze (day 16), open field test (half of the mice on day 19 and the other half of the mice on day 20) and tail suspension (day 22) tests (Figure 1).

### 2.4. Elevated plus Maze

The elevated plus maze apparatus consisted of two opposite open (17.5 × 5 cm) arms without sidewalls and two enclosed arms of the same size with 14 cm high sidewalls and an end wall. The apparatus was elevated to a height of 30 cm and placed in a room with a light intensity of 100 lx in open arms. Testing began by placing the animal on the central platform of the maze, facing an open arm. After each mouse, the floor of the testing apparatus was cleaned with 70% ethanol and dried thoroughly. A Standard 5 min test duration was employed, and all the sessions were video recorded. An arm entry was counted only when all four limbs were within a given arm.

### 2.5. Open Field Test

Locomotor activity of individual mice was measured with the illumination level of 450 lx for 30 min in soundproof photoelectric motility boxes (44.8 × 44.8 × 45 cm) connected to a computer (TSE, Technical & Scientific Equipment GmbH, Berlin, Germany). The floor of the testing apparatus was cleaned with 70% ethanol and dried thoroughly after each mouse. The system automatically registered the movement of the animal: the distance traveled, the number of rearings, corner visits, time spent, and distance covered in the central part of the box.

### 2.6. Tail Suspension Test

Mice were suspended for 6 min from the edge of a shelf 60 cm above a tabletop by adhesive tape, placed approximately 1 cm from the tip of the tail. The duration of immobility, the number of immobility episodes (an episode defined as hanging passively and being motionless for at least 3 s), and the number of short immobility episodes lasting 1–2 s were scored during the last 4 min from the recorded videos by an observer blind to the genotype.

### 2.7. Measurement of Monoamines

Immediately after the last behavioral test, all mice were decapitated. Brains were dissected into five parts and frozen in liquid nitrogen. Dorsal striatum (*caudate putamen*), ventral striatum (including *nucleus accumbens* and *olfactory tubercle*), hippocampus, and frontal cortex were dissected from the brains of all mice; from the brains of mice receiving chronic amphetamine (cohort Ib), ventral tegmental area, was dissected and from the mice receiving chronic escitalopram (cohort II), the raphe nuclei (including both dorsal and median groups of the raphe nuclei) were dissected. The brain dissection was performed according to the coordinates presented in the mouse brain atlas [49]. Monoamine measurements from the striatum were done differently compared to raphe nuclei and hippocampi. Monoamines–serotonin (5-HT), noradrenaline (NA) and dopamine (DA)–and their metabolites–5-hydroxyindoleacetic acid (5-HIAA), normetanephrine (NMN), 3,4-dihydroxyphenylacetic acid (DOPAC), homovanillic acid (HVA), and 3-methoxytyramine (3-MT)–were assayed by high-performance liquid chromatography (HPLC) with electrochemical detection. Monoamines and their metabolites were measured from the ventral and dorsal striatum (VSTR and DSTR) tissues of the mice from the chronic amphetamine experiment.

Monoamine quantification in raphe nuclei and hippocampi was carried out by liquid chromatography mass spectrometry. Briefly, the samples were weighed and transferred into 50 µL PBS. Fifty µl internal standard [^2^H_3_]leucine, [^13^C_6_]tyrosine, [^2^H_5_]phenylalanine (Cambridge Isotope Laboratories, Tewksbury, MA, USA), and 0.9–2.0 mm stainless steel beads (Next Advance, Troy, NY, USA) were added. Homogenization was achieved within 2 min in a bullet blender (Next Advance). Thereafter, 400 µL of ice-cold methanol (resulting the final concentration of 80% methanol) was added and the samples were left to stand at −20 °C for 20 min. After centrifugation for 10 min at 21,000× *g*, two 200 µL aliquots were taken from the supernatant and dried under a stream of nitrogen. The first aliquot was treated with 50 µL phenylisothiocyanate in water and pyridine (v/v/v 50/320/635) for 40 min at 40 °C. The second aliquot was treated with 100 µL 200 mM 2-nitrophenylhydrazine and 20 µL 120 mM 1(3-dimethylaminopropyl)-N-ethylcarbodiimide for 1 h at room temperature. After subsequent drying under nitrogen, the samples were resolved in 100 µL 5 mM ammonium acetate in methanol. Ten µl was injected into Acquity Premier 1.7 µm CSH Phenyl-Hexyl 2.1 × 100 mm column in Waters Acquity UPLC H-class–Xevo TQ-XS mass spectrometer (Waters, Milford MA, USA). The gradient was composed of solvent A: H_2_O with 0.2% formic acid and solvent B: acetonitrile with 0.2% formic acid. Starting from 85% solvent A for 0.5 min the gradient rose to 50% B in 1 min and to 90% B in the next 0.5 min. The total run time was 4.5 min with a flow rate 0.5 mL/min. The phenylisothiocyanate derivatives were analyzed in positive ionization multiple reaction monitoring modes with the following quantification ion pairs: 5-HT 312/160, NA 287/135, DA 289/137, NMN 301/166, 5-HIAA 192/146 and 3-MT 303/94. Nitrophenylhydrazine derivatives were quantified in negative ionization mode with the following ion pair signals: DOPAC 302/137, HVA 316/146.

### 2.8. Immunohistochemistry

To specify the location of NEGR1 in the monoaminergic pathways, anti-NEGR1 immunostaining was performed along with co-immunostainings specific for tyrosine hydroxylase and tryptophan hydroxylase. Fluorescent immunohistochemistry was performed on floating 30 µm thick coronal sections collected after every 300 μm into phosphate-buffered saline (PBS). Incubations were performed with gentle rocking and at room temperature unless mentioned otherwise. After washing with PBS for 10 min, sections were permeabilized with 0.25% Triton X-100 (Naxo, Tartu, Estonia)/PBS solution for 45 min. Sections were subsequently blocked in solution containing 0.3 M glycine/5% donkey serum//1% bovine serum albumin (BSA, Sigma-Aldrich)/PBS for 2 h at room temperature and incubated with rat anti-dopamine transporter/DAT (1:100, Santa Cruz Biotechnology, Heidelberg, Germany; sc-32258), sheep anti-TH (tyrosine hydroxylase) antibody (1:1000, Abcam, Cambridge, United Kingdom, Cat# ab113, RRID:AB_297905) in combinations with rabbit anti-TPH2 (tryptophan hydroxylase isoform 2) (1:500, Abcam Cat# ab26092, RRID:AB_2207690) and mouse anti-Negr1 antibody (1:50, Santa Cruz Biotechnology, H-12: sc-393293) dilutions in 0.1% Tween-20/1% BSA/PBS 72 h at 4 °C. Subsequently sections were then washed with 5 times in 0.1% Tween-20//PBS for 10 min and incubated with the appropriate secondary antibody Alexa Fluor^®^ 647 AffiniPure Donkey Anti-Rat IgG (H + L) (1:1000, Jackson ImmunoResearch Labs, West Grove, PA, USA; 712-607-003), FITC AffiniPure donkey anti-rabbit (1:1000, Jackson ImmunoResearch Lab., 711–095–152, RRID:AB_2315776), donkey anti-sheep IgG (H + L) Alexa Fluor 594 (1:1000, Thermo Fisher Scientific, Waltham, MA, USA, Cat# A11016, RRID:AB_10562537), donkey anti-mouse IgG (H + L) Alexa Fluor 647 (1:1000, Thermo Fisher Scientific, Cat# A-31571, RRID:AB_162542) at room temperature for 2 h. After subsequent washes with PBS 3 times for 10 min, the nuclei were stained with 5 μg/mL Bisbenzimide H 33258 (Hoechst 33258, Sigma Aldrich) in PBS for 2 min. Subsequently, sections were rinsed with PBS for 5 min, mounted in Fluoromount mounting medium (Sigma Aldrich), and covered with a 0.17 mm coverslip (Deltalab, Barcelona, Spain). The specificity of the immunohistochemistry was determined by incubations without the primary antibodies. Fluorescent images were obtained with the Olympus FV1200MPE (Olympus, Hamburg, Germany) laser scanning confocal microscope and Leica Aperio VERSA Brightfield, Fluorescence and FISH Digital Scanner.

The fluorescent intensity of dopamine transporter (DAT) immunostaining in the striatum was quantified using the Positive pixel count 2004-08-11 algorithm of Aperio Image Scope [v12.4.3.5008]. The image of the striatal surface was divided into DSTR containing caudate-putamen and VSTR consisting of nucleus accumbens and olfactory tubercle, according to the Scalable Brain Atlas [50]. Isosurface was created separately from both the parts of the striatum and was used to make quantitative measurements on area and surface fluorescent intensity.

### 2.9. RT-qPCR Analysis in Mouse Brain Areas

Gene expression was determined by two-step RT-qPCR (qPCR). Total RNA was extracted from each tissue sample by using Trizol reagent (Invitrogen) according to the manufacturer’s protocol. First-strand cDNA was synthesized by using FIREScript RT cDNA Synthesis MIX with Oligo (dT) and Random primers (Solis BioDyne, Tartu, Estonia) according to the manufacturer’s protocol.

In qPCR, 8 dopamine-related genes were studied, tyrosine hydroxylase (*Th*), dopamine receptor 1 (*Drd1*), dopamine receptor 2 (*Drd2*), dopamine receptor 5 (*Drd5*), dopamine transporter (*Dat*), catechol-O-methyltransferase (*Comt*), monoamine oxidase A (*MaoA*), and monoamine oxidase B (*MaoB*). Dopamine system-related primers used in the experiment have been previously described by Varul et al. [51]. Additionally, two serotonin system-related genes were studied: serotonin transporter (*Slc6a4*) and tryptophan hydroxylase (*Tph2*). As a housekeeper gene, beta-actin (ActB_mm_F ACCATGTACCCAGGCATTGC, ActB_mm_R AGCCACCGATCCACACAGAG) was used. Every reaction was made in four parallel replicates to minimize possible errors. All reactions were performed in a final volume of 10 μL, using 5 ng of cDNA. Real-time qPCR was performed using HOT FIREPol^®^ EvaGreen^®^ qPCR Supermix (Solis BioDyne). ABI Prism 7900HT Sequence Detection System with ABI Prism 7900 SDS 2.4.2 software (Applied Biosystems) was used for qPCR detection. qRT-PCR data in the Figures is presented on a linear scale, calculated as 2^−ΔΔCT^, where ΔCT is the difference in cycle threshold (CT) between the target genes and the housekeeper gene.

### 2.10. Statistical Analysis

Results are expressed as mean values ± SEM. The normal distribution of data was evaluated with the Shapiro–Wilk test. Results from qPCR and other data comparing two groups were assessed using Student’s *t*-test or Mann–Whitney test for nonparametric data. Detailed information about the analysis (normality estimates, statistical test used and *p*-values) of RT-qPCR data, comparing only two groups (WT and *Negr1*^−/−^), can be found in Supplementary Tables S1–S5. Comparison of RT-qPCR data from chronic amphetamine experiment in the VTA, of the behavioral results from the escitalopram experiment, levels of monoamines and their metabolites, and body weight differences were performed using two-way ANOVA followed by a Bonferroni post hoc test (Supplementary Table S6). The body weight dynamics from day -10 to day -3 were analyzed using repeated measures two-way ANOVA (time x genotype) followed by the Bonferroni post hoc test. Behavioral data from the chronic amphetamine experiment was analyzed by repeated measures three-way ANOVA followed by a Tukey’s post hoc test. All differences were considered statistically significant at *p* < 0.05. Statistical analysis was performed using GraphPad Prism 8 software.

## 3. Results

### 3.1. Negr1^−/−^ Mice Display Higher Sensitivity to Amphetamine Compared to WT Mice

**Figure 2:**
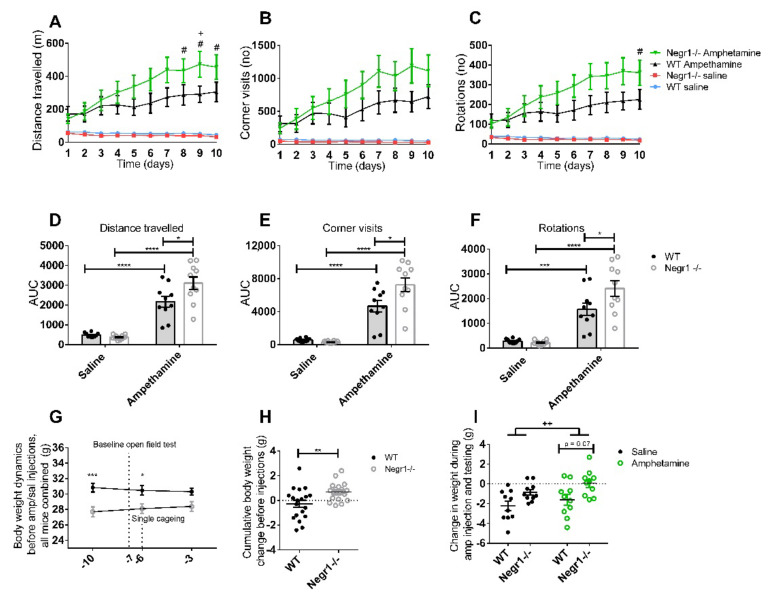
*Negr1*^−/−^ mice are more sensitive to chronic amphetamine administration. Effect of chronic amphetamine on (**A**,**D**) distance traveled, (**B**,**E**) corner visits, and (**C**,**F**) rotations in the open field test. In G and H saline/amphetamine groups have not been separated yet, the body weight change is a reaction to non-pharmacological environmental manipulations. (**G**) Body weight dynamics measured during 1 week of the period before amphetamine injection (from day -10 until day -3) (**H**). Cumulative body weight change before saline/amphetamine injections (from day -10 until day -3). (**I**) Body weight change caused by chronic saline or amphetamine injections and behavioral testing (from day 1 until day 10). Data represents mean ± SEM, +—*p* < 0.01—the difference in treatment in WT mice, #—*p* < 0.05—the difference in treatment in *Negr1*^−/−^ mice (Tukey post hoc test, (**A**–**C**)); ++—*p* < 0.01—genotype effect, * *p* < 0.05, ** *p* < 0.01, *** *p* < 0.001, **** *p* < 0.0001 (Mann–Whitney, (**D**–**F**); Bonferroni post hoc test, (**G**–**I**)). AUC, area under curve.

In the dose-response curve experiment, 6 mg/kg amphetamine-induced highly increased motor activity in both genotypes (Supplementary Figure S1A) and, therefore, the 6 mg/kg dose was considered to be too high for the chronic experiment, as the behavioral sensitization effect of amphetamine is well known [52]. Therefore, a chronic amphetamine experiment was performed by using a 3 mg/kg dose, which, if first time injected, induced only a tendency towards increased activity in batch 1a mice (Supplementary Figure S1A) but also statistically significant increase in the distance traveled in wt mice in bach 1b (*p* < 0.05, two-way ANOVA (Bonferroni post hoc test) (for more details, Supplementary Figure S1D and Figure legend). In order to assess the impact of amphetamine on behavior, *Negr1*^−/−^ and WT mice received 10 days of amphetamine in the dose of 3 mg/kg. The administration of amphetamine for 10 days induced significantly higher motor activity in *Negr1*^−/−^ mice compared to WT mice. In particular, amphetamine-treated *Negr1*^−/−^ mice had traveled, visited corners, and rotated more compared to WT mice, whereas the control (saline) groups for both genotypes did not show any differences in these activities (Figure 2A–F). Clockwise and anticlockwise rotations were summed up in these experiments as there was no difference in the direction of rotation. Three-Way Repeated-Measures ANOVA showed that distance traveled was affected by the time (F_3_._4,62_._6_ = 5.65; *p* = 0.001), treatment (F_1,18_ = 67.24; *p* < 0.0001), and genotype × treatment interaction (F_1,18_ = 22.84; *p* < 0.001) (Figure 2A, more details can be found in Supplementary Table S7). The number of corner visits showed significant time (F_3_._2,57_._9_ = 5.06; *p* = 0.002), treatment (F_1,18_ = 63.32; *p* < 0.0001), and genotype × treatment effects (F_1,18_ = 17.36; *p* < 0.001) (Figure 2B). Rotations were also affected by the time (F_3_._6,64_._8_ = 5.48; *p* = 0.001), treatment (F_1,18_ = 43.95; *p* < 0.0001) and genotype × treatment interaction (F_1,18_= 21.03; *p* < 0.001) (Figure 2C) (Supplementary Table S7).

Analyzing the AUC showed that amphetamine increased the traveled distance for both WT and *Negr1*^−/−^ mice (*p* < 0.0001; Figure 2D). Nevertheless, the traveled distance was longer in *Negr1*^−/−^ mice that received amphetamine compared to their WT littermates (*p* = 0.016; Figure 2D). A similar situation was also seen in the case of corner visits (Figure 2B,E) and rotations (Figure 2C,F): while both genotypes visited more corners as well as made more rotations (*p* < 0.001 for WTs and *p* < 0.0001 for *Negr1*^−/−^ mice), (Figure 2E) upon chronic amphetamine administration. Besides that, in summary, the amphetamine-induced more corner visits (*p* = 0.012) and rotations (*p* = 0.032) in *Negr1*^−/−^ mice compared to their WT littermates.

The body weight of the mice was measured 10 days before amphetamine or saline injections (marked as day -10). Body weight dynamics prior to the amphetamine injection (day -10 until -3) showed significant genotype (F_1,38_ = 9.17; *p* = 0.004) and genotype x time interaction effects (F_2, 76_ = 5.97; *p* = 0.004). WT mice showed a slight decrease in body weight, whereas *Negr1*^−/−^ mice gained weight during the first week of measurements (Figure 2G) at the time when handling due to daily weighing was the only interfering activity. According to Bonferroni’s post hoc test on day -10 the difference between the body weight of the WT mice and *Negr1*^−/−^ mice was statistically significant (*p* = 0.001). On day -7, the baseline open field test was performed, and on day -6 all the mice were single housed. The difference in body weight was still statistically significant on day -6 (*p* = 0.018) but was not seen anymore on day -3. The change in body weight during the first week (days -10 vs. day -3) was also calculated (Figure 2H). The average weight loss for WT mice was 0.27 g, and the average weight gain of *Negr1*^−/−^ mice was 0.69 g. Mann–Whitney test showed that weight change differences between WT and *Negr1*^−/−^ mice were statistically significant (*p* = 0.008). The weight from the beginning of the experiment to the end of the experiment (day -10 vs. day 10) was also calculated (Figure 2I), but there were no statistically significant changes in the body weight change caused by amphetamine. There was, however, a significant genotype effect (F_1,36_ = 11.62; *p* = 0.002) showing that *Negr1*^−/−^ mice lost less body weight than WT both upon saline and amphetamine injections.

If the data from saline groups were analyzed separately (Supplementary Figure S2), genotype differences were evident in the distance traveled in the center and rotations, as well as in the number of rearings in the open field test. The post hoc test showed no significant changes between *Negr1*^−/−^ and WT mice for individual days. For each mouse, the AUC was calculated, and the results analyzed using the Mann–Whitney U test showed that *Negr1*^−/−^ mice performed significantly fewer rearings compared to WT mice (*p* = 0.012). The AUC of distance traveled in the center of the box was significantly lower in *Negr1*^−/−^ mice (*p* = 0.035).

### 3.2. Chronic Administration of Amphetamine Increases the Level of Tyrosine Hydroxylase (TH) in VTA

**Figure 3:**
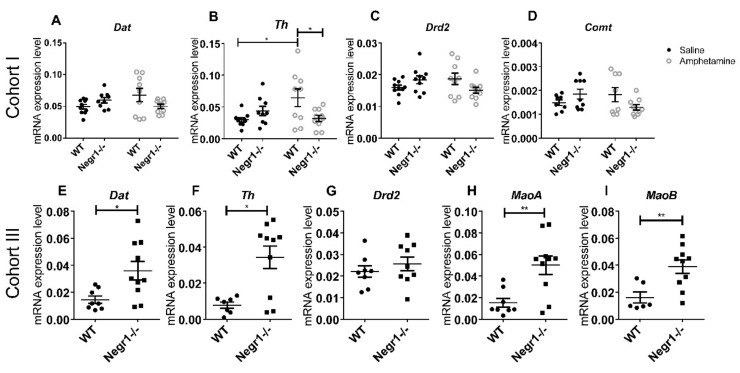
The level of dopamine system-related genes in the VTA of mice. Relative mRNA expression levels of (**A**) dopamine transporter (*Dat*), (**B**) tyrosine hydroxylase (*Th*), (**C**) dopamine receptor D2 (*Drd2*), (**D**) catechol-*O*-methyltransferase (*Comt*) in *Negr1*^−/−^ mice and their WT littermates after 10 days of chronic saline or amphetamine i.p. injection (cohort I). The levels of (**E**) dopamine transporter (*Dat*), (**F**) tyrosine hydroxylase (*Th*), (**G**) dopamine receptor D2 (*Drd2*), (**H**) monoamine oxidase A (*MaoA*) and (**I**) monoamine oxidase B (*MaoB*) in home-cage *Negr1*^−/−^ mice and their WT littermates (cohort III). Data represents mean ± SEM, * *p* < 0.05, ** *p* < 0.01, ordinary two-way ANOVA (Bonferroni post hoc test) (**A**–**D**), Mann–Whitney U test (**E**–**I**).

To assess the level of dopamine system-related genes in VTA, qPCR was performed using both cohort I (Figure 3A–D) and cohort III mice (Figure 3I). There was a genotype × treatment interaction (F_1,34_ = 8.3; *p* = 0.007) effect on the level of *Th* in the VTA. Post hoc tests showed that chronic amphetamine treatment significantly increased the level of *Th* only in WT mice (*p* = 0.027). The level of *Th* was significantly higher in the WT amphetamine group compared to the *Negr1*^−/−^ amphetamine group (*p* = 0.045) (Figure 3B). Post hoc analysis confirmed no other significant changes in the level of dopamine system-related genes *(Dat*, *Drd2*, and *Comt*) between the groups in the VTA of cohort I mice (Figure 3A,C,D). The detailed results of ANOVA have been shown in Supplementary Table S6.

Mann–Whitney U Test or *t*-test (according to normality distribution) was used to compare WT and *Negr1*^−/−^ groups, statistical details can be found in Supplementary Table S1. In the VTA, the level of *Dat* was significantly higher in *Negr1*^−/−^ mice (*p* = 0.011) (Figure 3E). The levels of *Th* (*p* = 0.019) (Figure 3F), *MaoA* (*p* = 0.009) (Figure 3H), and *MaoB* (*p* = 0.005) (Figure 3I) were also significantly higher in the VTA of *Negr1*^−/−^ mice compared to WT mice. There were no significant changes in the level of *Drd2* in the VTA of cohort II mice (Figure 3G).

### 3.3. Amphetamine Increases the Level of Dopamine in Dorsal Striatum (DSTR)

**Figure 4:**
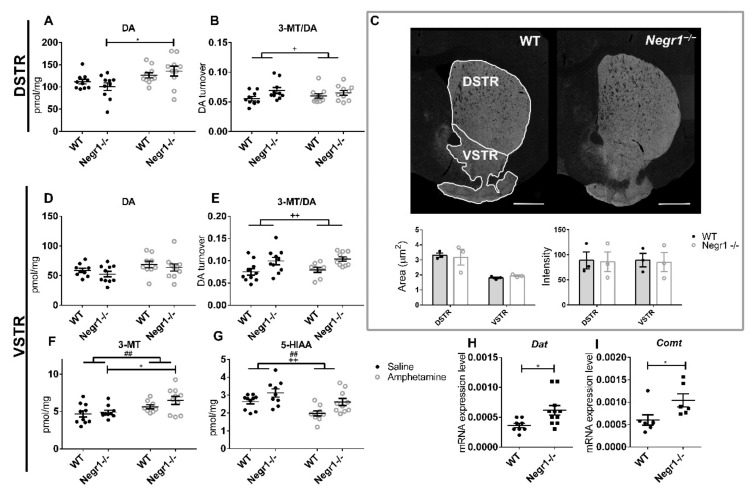
Effect of chronic amphetamine in the dorsal striatum (DSTR) and ventral striatum (VSTR). The level of (**A**) dopamine (DA) and (**B**) dopamine turnover (3-MT/DA) in the DSTR. (**C**) Immunohistochemical DAT stainings of DSTR and VSTR. The level of (**D**) dopamine (DA), (**E**) dopamine turnover (3-MT/DA), (**F**) 3-MeOTyramine (3-MT), (**G**) 5-Hydroxyindoleacetic acid (5-HIAA), (**H**) dopamine transporter (*Dat*), and (**I**) catechol-*O*-methyltransferase (*Comt*) in the VSTR. Data represent mean ± SEM, ## *p* < 0.01—treatment effect, + *p* < 0.05, ++ *p* < 0.01—genotype effect, * *p* < 0.05—post hoc test, ordinary two-way ANOVA (Bonferroni post hoc test), Mann–Whitney U test (**H**,**I**), WT – wild-type.

To identify the differences caused by chronic amphetamine administration between WT and *Negr1*^−/−^ two-way ANOVA (treatment (amphetamine or saline) × genotype (WT or *Negr1*^−/−^)) and Bonferroni post hoc test was used. The level of dopamine and its metabolites were measured in DSTR (Supplementary Tables S2 and S8; Figure S3) and VSTR (Supplementary Table S9). In the DSTR, the level of DA was affected by the treatment (treatment: F_1,36_ = 9.19; *p* = 0.005; Bonferroni’s post hoc tests showed that chronic treatment with amphetamine significantly increased the level of DA in *Negr1*^−/−^ (*p* = 0.026) but not in control mice (Figure 4A). Dopamine turnover (3-MT/DA) in the DSTR was affected by the genotype (F_1,36_ = 5.30; *p* = 0.027) (Figure 4B), post hoc test showed no statistically significant changes between the groups. Immunohistochemical DAT staining of DSTR and VSTR did not show any significant differences between WT and *Negr1*^−/−^ mice (Figure 4C).

In the VSTR, the level of DA displayed no significant changes between the groups (Figure 4D, Supplementary Table S9); whereas, dopamine turnover to 3-MT (3-MT/DA) was again affected by the genotype (F_1,34_ = 12.51; *p* = 0.001) (Figure 4E). The level of DA metabolite 3-MT itself was affected by the treatment (F_1,34_ = 9.77; *p* = 0.004) (Figure 4F), and post hoc test revealed that the level of 3-MT was significantly increased in the *Negr1*^−/−^ group receiving amphetamine (*p* = 0.047) in comparison to the saline-injected *Negr1*^−/−^. The level of serotonin metabolite 5-HIAA was significantly affected by both treatment (F_1,34_ = 10.67; *p* = 0.003) and genotype (F_1,34_ = 10.06; *p* = 0.003) (Figure 4G, Supplementary Table S9). In the VSTR, the levels of DA system-related genes *Dat* (*p* = 0.011) (Figure 4H) and *Comt* (*p* = 0.014) (Figure 4I) were significantly higher in *Negr1*^−/−^ mice compared to WT mice (Supplementary Table S3).

Amphetamine reduced the turnover of DA to DOPAC and DA to HVA (Supplementary Figure S3). In the dorsal striatum, the levels of DOPAC (F_1,35_ = 38.61; *p* < 0.0001, DOPAC/DA (F_1,35_ = 45.23; *p* < 0.0001, HVA (F_1,35_ = 90.40; *p* < 0.0001 were affected by treatment. The ratio HVA/DA was significant if genotype (F_1,34_ = 6.870; *p* = 0.013) and treatment (F_1,34_ = 100.6; *p* < 0.0001) were considered. In the ventral striatum, the level of DOPAC (F_1,36_ = 9.19; *p* = 0.0045), DOPAC/DA (F_1,36_ = 136.2; *p* < 0.0001), HVA (F_1,36_ = 75.26; *p* < 0.0001), and HVA/DA were affected by treatment (F_1,36_ = 92.09; *p* < 0.0001).

**Figure 5:**
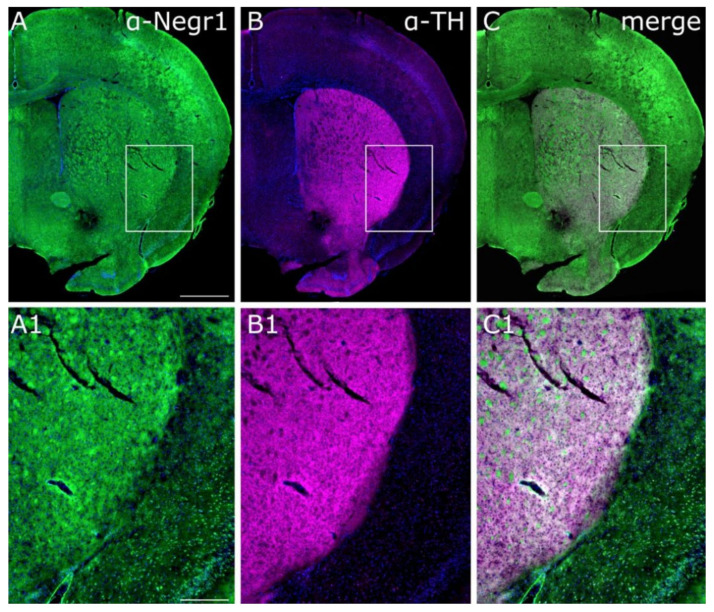
Expression of NEGR1 and TH in the striatum. Representative confocal images of the striatum show co-immunohistochemical stainings of anti-NEGR1 in green (**A**,**A1**), with anti-tyrosine hydroxylase in magenta (TH) (**B**,**B1**) and NEGR1 staining in the vicinity of dopaminergic projection area can be seen with white color merged images (**C**,**C1**). Boxed areas show the localization of the close-ups in the images (**A1**–**C1**). Scale bars: (**A**–**C**) 1 mm, (**A1**–**C1**) 300 µm. LV—lateral ventricles, Ctx—central cortex, Sept—septum, CPu—caudate putamen, NAc—nucleus accumbens, OT—olfactory tubercule, Pir—piriform cortex.

In the striatum of WT mice, NEGR1 antibody gave a signal in the vicinity of the dopaminergic projection area, which was marked with anti-tyrosine hydroxylase (TH)—the rate-limiting enzyme of dopamine production (Figure 5A–C,A1–C1).

### 3.4. Chronic Amphetamine Administration Alters the Level of Monoamines in the Hippocampus and Chronic Escitalopram Treatment Causes Weight Difference in the Hippocampus

**Figure 6:**
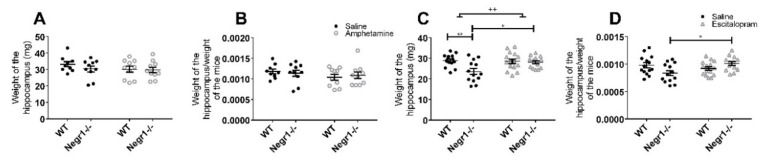
Hippocampi of *Negr1*^−/−^ mice weigh less compared to WT mice, and escitalopram restores the weight of the hippocampi of *Negr1*^−/−^ mice. (**A**) The weight of the hippocampi of the mice after receiving 10 days of saline or amphetamine. (**B**) Weight of hippocampi divided by the weight of mice (after receiving 10 days of saline or amphetamine). (**C**) Weight of hippocampi of the mice receiving 23 days of saline or escitalopram. (**D**) Weight of hippocampi divided by the weight of mice (after receiving 23 days of saline or escitalopram). Data represent mean ± SEM, ++ *p* < 0.01—genotype effect, * *p* < 0.05, ** *p* < 0.01—post hoc test, ordinary two-way ANOVA (Bonferroni post hoc test).

Hippocampi of the *Negr1*^−/−^ mice weigh less compared to WT hippocampi (Figure 6C,D). Amphetamine had no effect on the hippocampal weights of either *Negr1*^−/−^ or WT mice (Figure 6A,B). In the escitalopram treatment experiment, the weight of hippocampi was significantly affected by the genotype (F_1,51_ = 7.38; *p* = 0.009), and there was a significant genotype x treatment interaction (F_1,51_ = 5.06; *p* = 0.03). The post hoc test showed that hippocampi of the *Negr1*^−/−^ mice that received saline weighed less compared to WT saline group mice hippocampi (*p* = 0.006) and *Negr1*^−/−^ escitalopram group hippocampi weighed significantly more compared to *Negr1*^−/−^ saline group hippocampi (*p* = 0.03) (Figure 6C). If the weight of hippocampi was divided by the body weight of mice, it was seen that the hippocampi/body weight relationship was affected by genotype × treatment interaction (F_1,51_ = 8.01; *p* = 0.007). The post hoc test showed that this relationship was significantly higher in the *Negr1*^−/−^ escitalopram group compared to the *Negr1*^−/−^ saline group (*p* = 0.03) (Figure 6D). On the other hand, chronic administration of either amphetamine or escitalopram does not have an effect on the body weight of mice (Supplementary Figures S4 and S5).

**Figure 7:**
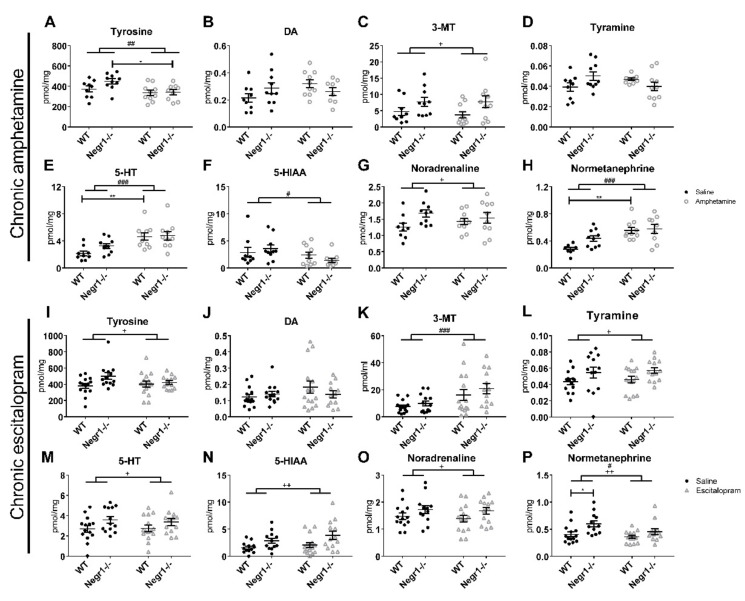
Effect of chronic administration of amphetamine or escitalopram on the level of monoamines and their metabolites in the hippocampus. The levels of (**A**) tyrosine, (**B**) dopamine (DA), (**C**) 3MeOTyramine (3-MT), (**D**) tyramine, (**E**) serotonin (5-HT), (**F**) 5-hydroxyindoleacetic acid (5-HIAA), (**G**) noradrenaline and (**H**) normetanephrine in the hippocampus of *Negr1*^−/−^ mice and their WT littermates after 10 days of chronic amphetamine i.p. injections. The levels of (**I**) tyrosine, (**J**) dopamine (DA), (**K**) 3MeOTyramine (3-MT), (**L**) tyramine, (**M**) serotonin (5-HT), (**N**) 5-Hydroxyindoleacetic acid (5-HIAA), (**O**) noradrenaline and (**P**) normetanephrine in the hippocampus of *Negr1*^−/−^ mice and their WT littermates after 23 days of chronic escitalopram i.p. injections. Data represent mean ± SEM, # *p* < 0.05, ## *p* < 0.01, ### *p* < 0.001- treatment effect, + *p* < 0.05, ++ *p* < 0.01—genotype effect, * *p* < 0.05, ** *p* < 0.01—post hoc test, ordinary two-way ANOVA (Bonferroni post hoc test).

The level of monoamines and their metabolites were measured in the hippocampi of mice receiving either chronic treatment of amphetamine (Supplementary Table S10) (Figure 7A–H) or escitalopram (Supplementary Table S11) (Figure 7I–P). In the case of chronic amphetamine administration, the level of tyrosine (Tyr) was affected by the treatment (F_1,34_ = 7.78; *p* = 0.009). Bonferroni’s post hoc test revealed that the level of tyrosine was significantly decreased by amphetamine in the *Negr1*^−/−^ group (*p* = 0.039) (Figure 7A). The level of 3-MT was affected by the genotype (F_1,35_ = 6.00; *p* = 0.019) (Figure 7C) and the level of tyramine was affected by the genotype x treatment interaction (F_1,35_ = 5.82; *p* = 0.021) (Figure 7D), but post hoc test showed no significant changes in case of neither of them. The level of 5-HT was affected by the treatment (F_1,35_ = 17.51; *p* < 0.001), the post hoc test showed that amphetamine increased the level of 5-HT in the WT mice group (*p* = 0.005) (Figure 7E). There was a treatment effect (F_1,35_ = 4.18; *p* = 0.049) on the level of 5-HIAA (Figure 7F), the post hoc comparison did not indicate significant differences in *Negr1*^−/−^ or WT mice separately. The level of NA was affected by the genotype (F_1,35_ = 4.36; *p* = 0.044) (Figure 7G) and its metabolite NMN was affected by the treatment (F_1,33_ = 19.44, *p* < 0.001), post hoc test showed that amphetamine increased the level of NMN of the WT mice (*p* = 0.002) (Figure 7H).

In the chronic escitalopram experiment, there was a genotype effect (F_1,51_ = 4.25; *p* = 0.045) on the level of Tyr (Figure 7I, Supplementary Table S10); Bonferroni’s post hoc test showed no significant changes. The level of 3-MT was affected by the treatment (F_1,51_ = 10.36; *p* = 0.002) (Figure 7K). The change of levels of tyramine (F_1,51_ = 5.93; *p* = 0.018) (Figure 7L), 5-HT (F_1,51_ = 5.40; *p* = 0.024) (Figure 7M), 5-HIAA (F_1,49_ = 8.64; *p* = 0.005) (Figure 7N) and NA (F_1,50_ = 4.14; *p* = 0.047) (Figure 7O) were affected by the genotype. The level of NMN was affected by both the treatment (F_1,50_ = 4.57; *p* = 0.038) and genotype (F_1,50_ = 10.55; *p* = 0.002); the post hoc test showed that the level of NMN was higher in the *Negr1*^−/−^ saline group compared to WT saline group (*p* = 0.023) (Figure 7P).

### 3.5. Chronic Administration of Escitalopram Alters the Level of Monoamines and Their Metabolites in Raphe

**Figure 8:**
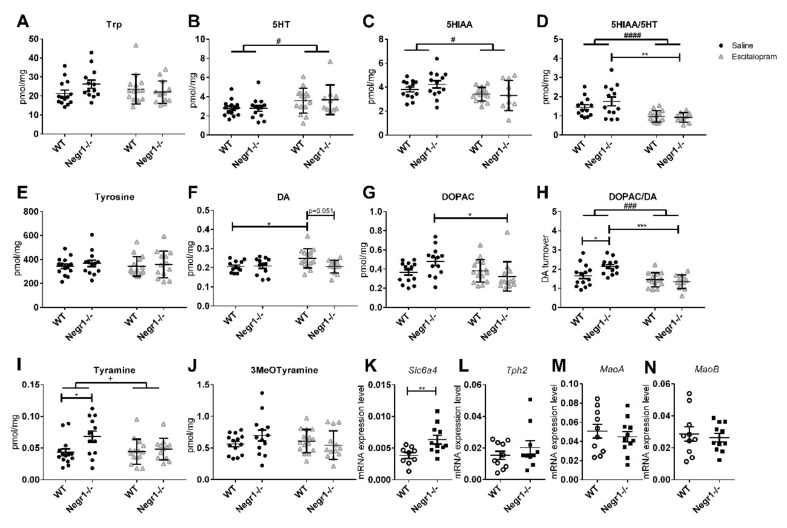
Effect of chronic escitalopram on the level of monoamines and their metabolites in the raphe nuclei. Batch II mice (**A**–**J**) and batch III mice (**K**–**N**). Levels of (**A**) tryptophan (Trp), (**B**) serotonin (5-HT), (**C**) 5-Hydroxyindoleacetic acid (5-HIAA), (**D**) serotonin turnover (5-HIAA/5-HT), (**E**) tyrosine, (**F**) dopamine (DA), (**G**) 3,4-Dihydroxyphenylacetic acid (DOPAC), (**H**) dopamine turnover (DOPAC/DA), (**I**) tyramine and (**J**) 3MeOTyramine in raphe. The mRNA expression level of (**K**) serotonin transporter (*Slc6a4*), (**L**) tryptophan hydroxylase 2 (*Tph2*), (**M**) monoamine oxidase A (*MaoA*), and (**N**) monoamine oxidase B (*MaoB*). Data represent mean ± SEM, # *p* < 0.05, ### *p* < 0.001, #### *p* < 0.0001—treatment effect, + *p* < 0.05—genotype effect, * *p* < 0.05, ** *p* < 0.01, *** *p* < 0.001—post hoc test, ordinary two-way ANOVA (Bonferroni post hoc test) (A-J), Mann–Whitney U test (K-N). WT- wild-type.

Levels of different monoamines and their metabolites were measured in the raphe nuclei (Figure 8A–J) (Supplementary Table S12). In the raphe, the levels of 5-HT (F_1,48_ = 6.36; *p* = 0.015) (Figure 8B) and its metabolite 5-HIAA (F_1,47_ = 6.23; *p* = 0.016) (Figure 8C) were affected by the treatment, but Bonferroni’s post hoc test did not show any significant changes. In the raphe, escitalopram significantly decreased the 5-HT turnover (5-HIAA/5-HT) in the *Negr1*^−/−^ group (*p* = 0.002) (Figure 8D), 5-HT turnover was affected by the treatment (F_1,46_ = 19.11; *p* < 0.0001). The level of tyrosine showed no statistically significant differences between the groups (Figure 8E).

The level of DA was affected by treatment × genotype interaction (F_1,47_ = 4.11; *p* = 0.048). Escitalopram significantly increased the level of DA in the WT group (*p* = 0.048) but not in *Negr1*^−/−^ mice (Figure 8F). The level of DA metabolite DOPAC was also affected by the treatment x genotype interaction (F_1,51_ = 5.80; *p* = 0.02), the level of DOPAC was statistically significantly higher in the *Negr1*^−/−^ group receiving saline, compared to the *Negr1*^−/−^ escitalopram group (*p* = 0.025) (Figure 8G). There was a treatment (F_1,49_ = 17.53; *p* < 0.001) and treatment x genotype interaction (F_1,49_ = 6.03; *p* = 0.018) effect on DA turnover (DOPAC/DA). DA turnover was significantly higher in the *Negr1*^−/−^ saline group compared to the WT saline group (*p* = 0.035), and escitalopram significantly decreased the DA turnover in the *Negr1*^−/−^ group (*p* < 0.0001) (Figure 8H). There was a significant genotype effect on the level of tyramine (F_1,49_ = 5.38; *p* = 0.025). The level of tyramine was statistically significantly higher in the *Negr1*^−/−^ saline group compared to the WT saline group (*p* = 0.043) (Figure 8I). There were no significant changes in the level of 3-MT (Figure 8J).

The serotonin system-related genes were measured in the raphe using qPCR. Mann–Whitney U Test or *t*-test (according to normality distribution) was used to compare WT and *Negr1* groups, statistical details can be found in Supplementary Table S4. (Figure 8K–N). In the raphe, the level of *Slc6a4* was significantly higher in *Negr1*^−/−^ mice (*p* = 0.009) (Figure 8K). There were no significant differences between *Negr1*^−/−^ and WT mice in the level of *Tph2* (Figure 8L), *MaoA* (Figure 8M), and *MaoB* (Figure 8N). The levels of dopamine system-related genes were found to be unaltered in the frontal cortex (Supplementary Table S5). In the DSTR, the level of *Dat* showed a significant difference between *Negr1*^−/−^ mice compared to WT mice, *p* = 0.029; the details of statistical analysis and *p*-values can be found in Supplementary Table S2.

**Figure 9:**
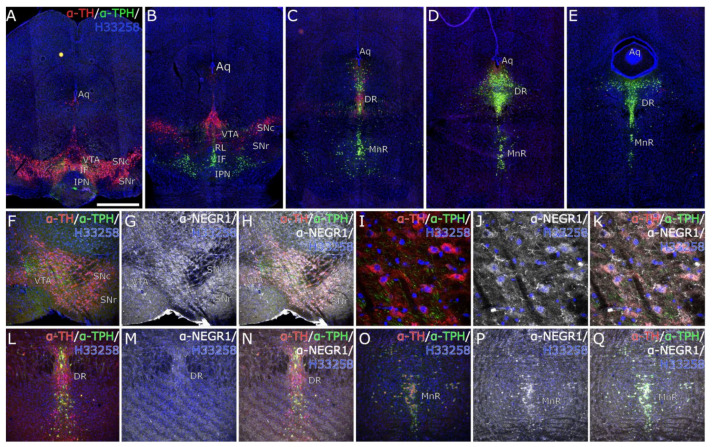
Immunohistochemical staining of WT mouse brain coronal sections displaying an expression of tyrosine hydroxylase (TH), tryptophan hydroxylase 2 (TPH) with NEGR1. (**A**–**E**) epifluorescent images display localization of TH and TPH throughout dopaminergic and raphe nuclei. (**F**–**K**) laser scanning confocal microscope images displaying localization of NEGR1 in TH-positive cells in substantia nigra pars reticulata (SNr). (**L**–**N**) laser-scanning confocal microscope images show diffuse localization of NEGR1 in the dorsal raphe (DR), whereas (**O**–**Q**) the localization in medial raphe (MnR) is observable in cells expressing TH and TPH. (**A**–**Q**) Nuclei were stained using H33258 stain (blue). Scale bars: (**A**–**E**) 1 mm, (**F**–**H**,**L**–**Q**) 0.5 mm, (**I**–**K**) 100 µm. Aq—aqueduct, VTA—ventral tegmental area, IF—interfascicular nucleus, IPN—interpeduncular nucleus, SNc—substantia nigra pars compacta, SNr—substantia nigra pars reticulate, RL—rostral linear nucleus, DR—dorsal raphe nucleus, MnR—median raphe nucleus.

Triple immunohistochemical stainings of WT mice midbrain to pons area revealed expression of tyrosine hydroxylase (TH) and tryptophan hydroxylase (TPH), the rate limiting enzyme of serotonin production, throughout the dopaminergic and serotonergic neurons (Figure 9A–E). Localization of NEGR1 is also observed in cells expressing TH in substantia nigra pars reticulata and TPH in dorsal, median raphe, indicating the involvement of NEGR1 in both dopaminergic as well as serotonergic neurotransmission (Figure 9F–Q). The Supplementary Figure S4 presents specificity of Alexa 647 secondary antibody binding.

### 3.6. Chronic Administration of Escitalopram Causes No Alterations in the Behavior of Negr1^−/−^ Mice

*Negr1*^−/−^ and WT received 23 days of escitalopram, and the behavior of the mice was assessed in the elevated plus maze (day 16), open field test (days 19 and 20), and tail suspension test (day 22). The test results showed that in the tail suspension test latency-to-freeze was affected by the treatment (F_1,50_ = 4.189; *p* = 0.046). In the elevated plus maze test frequency of entering closed arms was affected by the genotype (F_1,50_ = 4.713; *p* = 0.035). In the open field test corner visits made within the first 5 min of the experiment was affected by the genotype (F_1,50_ = 4.356; *p* = 0.042). There were no other significant behavioral changes in the chronic escitalopram treatment experiment (Supplementary Figure S7 and Supplementary Table S13).

## 4. Discussion

### 4.1. NEGR1 Expression in Monoaminergic Brain Circuits

In human GWAS studies, the neural adhesion molecule encoding the *NEGR1* gene has been linked to both depression and obesity [1,3,6,7]. Altered monoaminergic neurotransmission has also been linked to both obesity [53,54] and depression [33], and these conditions have been associated with neural pathways that are guided and maintained in the presence of NEGR1 protein. The expression of *Negr1* has been shown in both dopaminergic nuclei and projection areas such as substantia nigra pars compacta, VTA, islands of Calleja in the VSTR [27] and in the fasciculus retroflexus, which serves as a molecular scaffold for dopaminergic axons that grow from the midbrain towards the habenula [34]. Additionally, *Negr1* has been identified as a differentially expressed gene across 5-HT neuron subtypes, whereas the expression of *Negr1* was highest in the median raphe [36].

The current study is the first to explore the brain monoaminergic system in NEGR1-deficient mice to gain novel insights into whether these neural circuits could be responsible for the link between NEGR1 polymorphisms and phenotypes of depression and obesity. We challenged the monoaminergic neurotransmission of mice lacking *Negr1* and their WT littermates with a chronic injection of either amphetamine or escitalopram. The behavior of these mice was tested, and brain monoamines and gene expression were measured from the brain consequently. In our earlier studies, we have shown by using in situ hybridization that compared to other brain areas, the mRNA expression of *Negr1* is sparse in the striatal areas, especially in the DSTR of adult mice [55]. To estimate NEGR1 protein expression and impact on the dopaminergic signaling in the striatal area, we performed NEGR1 and tyrosine hydroxylase co-staining in the striatal area (Figure 5). We found that *Negr1* in the striatum is highly expressed in the fibers and moderately on cell bodies where *Negr1* also shows co-localization with tyrosine hydroxylase, indicating that *Negr1* is expressed in the same cells where dopamine is synthesized. The staining indicates that at least some amount of the NEGR1 protein in the striatum is not synthesized on the cell bodies in the striatum but is expressed on the axon bundles projecting through it. The more specific identification of these bundles remains to be clarified in future studies.

Next, we also studied the potential expression of NEGR1 in the region from the midbrain to pons by using triple immunostainings for tryptophan hydroxylase, tyrosine hydroxylase, and NEGR1 (Figure 9. Additionally, to the striatum, we found co-expression of tyrosine hydroxylase and NEGR1 also in the VTA/substantia nigra region. Our findings of more prevalent NEGR1 expression in the median raphe compared to the dorsal raphe are in line with the findings from Okaty et al. [36]. In the median raphe, NEGR1 is present in both the tyrosine hydroxylase and tryptophan hydroxylase-positive cells, whereas, in the dorsal raphe, the expression of NEGR1 is minor. In conclusion, we show that NEGR1 protein is present in both dopaminergic and serotonergic pathways; in the tyrosine hydroxylase-positive cells in both striatum and midbrain, and in the raphe where NEGR1 is mostly present in the median raphe. Considering our current knowledge about the function of NEGR1 in the nervous system [56], we propose that NEGR1 plays a role in the organization of protein networks at the monoaminergic synaptic cleft and/or in the regulation of motility and assembly of synaptic vesicles.

### 4.2. Increased Behavioral Sensitization to Amphetamine and Upregulation of Dat Transcript in Negr1^−/−^ Mice

In the current study, 10-day administration of amphetamine-induced significantly higher motor and stereotypic activity in *Negr1*^−/−^ mice compared to WT mice, indicating a higher sensitivity to amphetamine. It has been shown earlier that time-dependent changes in behavioral sensitization to amphetamine are associated with time-dependent changes in amphetamine-stimulated DA release in the striatum [57]. In our current study, significantly increased DA levels after 10-day administration of amphetamine were evident in the dorsal striatum in *Negr1*^−/−^ mice. However, the DA metabolite 3-MT was elevated in the ventral striatum in *Negr1*^−/−^ mice, indicating that DA release was increased there as well. Increased levels of *Comt* transcript in the ventral striatum is further supporting higher dopamine release along with higher DA turnover in the ventral striatum in *Negr1*^−/−^ mice. The difference that we see between the dorsal and ventral striatum could indicate the differential effect of amphetamine on dopamine release and uptake in the dorsal and ventral striatum [58,59].

Transcripts encoding proteins regulating dopaminergic neurotransmission were mostly significantly increased in the striatum and VTA area of *Negr1*^−/−^ mice. Namely, dopamine transporter (*Dat*) transcripts were significantly upregulated both in the VTA and ventral striatum in *Negr1*^−/−^ mice. Transcripts encoding tyrosine hydroxylase, *MaoA* and *MaoB* were upregulated in the VTA, whereas *Comt* was upregulated in the ventral striatum (Figure 3 and Figure 4). DAT plays a central role in the regulation of dopaminergic signaling; DAT overexpressing transgenic mice demonstrates markedly increased locomotor responses to amphetamine compared with WT animals [60]. Likewise, reduced DAT expression has been shown to diminish amphetamine’s locomotor stimulatory effects [61]. Furthermore, similarly to our current finding in the ventral striatum in *Negr1*^−/−^ mice, an increase in the amount of DA released by amphetamine has been shown in the DAT overexpressing mice [60].

Our previous studies have shown that deletion of other IgLONs, both *Lsamp* and *Ntm*, which are closely related NEGR1 homologs, are causing reduced sensitivity for acute amphetamine administration in mice [24,40]. Moreover, this phenotype is basically the only overlapping phenotype in mice deficient for either *Lsamp* or *Ntm* and the insensitivity for amphetamine is magnified in *Lsamp*^−/−^*Ntm*^−/−^ double mutant mice. Indeed, in the current study, we could not see clear genotype differences in amphetamine treatment groups; however, our data indicate a tendency toward reduced sensitivity in the case of acute administration of amphetamine. Therefore, members of the IgLON family of neural adhesion molecules could be collectively responsible for the fine-tuning of neural circuits involved in both acute and chronic responses to amphetamine.

### 4.3. Altered Molecular Reactivity to the Amphetamine in the Brains of Negr1^−/−^ Mice

The robust effect of amphetamine was similar in the striatum in both *Negr1*^−/−^ mice and their WT controls. Amphetamine reduced the turnover of dopamine to DOPAC and dopamine to HVA at a similar rate. Higher amphetamine-induced dopamine levels in the dorsal striatum and higher 3-MT levels in the ventral striatum were induced by amphetamine only in the *Negr1*^−/−^ mice. Increased DAT and tyrosine hydroxylase in the midbrain after amphetamine have been described earlier in wild-type animals [62,63], likewise, in the current study, amphetamine induced an increase in tyrosine hydroxylase and a trend towards increased levels of *Dat*, *Comt*, and *Drd2* in WT mice in the VTA. In *Negr1*^−/−^ mice, on the contrary, these transcripts showed a trend towards amphetamine-induced reduction, indicating altered molecular reactivity to amphetamine in the brains of *Negr1*^−/−^ mice. Similarly, in the hippocampi of WT mice, amphetamine significantly increased the levels of serotonin and normetanephrine and induced a tendency for an increase of several monoamines, including dopamine. In *Negr1*^−/−^ mice, the amphetamine-induced changes in the monoamine profile were quite different in the hippocampus, while the only significant effect of amphetamine was reduced levels of tyrosine (Figure 7). We also explored the effect of acute and chronic amphetamine on the NEGR1 transcript in various brain areas; however, the downregulation of *Negr1* transcript induced by chronic amphetamine was present only in the frontal cortex and only in 129Sv mice (Supplementary Figure S5).

The baseline levels of DA itself were not found to be altered in the brain areas of *Negr1*^−/−^ mice, however, several significant alterations in several brain areas of the *Negr1*^−/−^ mice suggest increased turnover of dopamine but also of serotonin. Increased turnover of DA to 3-MT was evident in both the dorsal and ventral striatum. Interestingly, serotonin metabolite 5-HIAA was increased only in the ventral striatum of *Negr1*^−/−^ mice. Although amphetamine suppressed 5-HIAA in both genotypes, the 5-HIAA still remained higher in *Negr1*^−/−^ mice. The upregulation or the tendency for upregulation of monoamines and their metabolites in saline-injected *Negr1*^−/−^ mice was most evident in the hippocampal area. The results from mice receiving saline chronically from the amphetamine study and escitalopram study could be regarded as replicates (Figure 6), and they indicate genotype differences between knockout and control groups in two distinct age groups; the age of mice at the end of escitalopram study was 3 months and the age of mice in the end of amphetamine study was 5 months. Significantly higher levels in *Negr1*^−/−^ mice compared to WT could be detected for tyrosine, 3-MT, tyramine, 5-HT, 5-HIAA, noradrenaline, and normetanephrine.

The stronger behavioral effect of amphetamine in *Negr1*^−/−^ mice could have also been modulated by the trace amine-associated receptor 1 (TAAR1), which has been shown to serve as a direct intracellular target for amphetamines in dopaminergic neurons [64]. TAAR1 is stimulated by amphetamine, but also by a variety of trace amines and monoamines, which are upregulated in the brains of *Negr1*^−/−^ mice, such as tyramine and 3-MT. The increased levels of endogenous agonists could have an impact on the sensitivity of TAAR1, which could, in turn, influence the effects of amphetamine.

Taken together, while the robust effect of amphetamine in the reduction of dopamine turnover in the striatum was similar in both genotypes, there were significant alterations in response to amphetamine in the brains of *Negr1*^−/−^ mice which indicated higher tone of dopaminergic neurotransmission in the dorsal striatum but blunted the response of dopamine system-related gene expression in the midbrain.

### 4.4. Negr1^−/−^ Mice Display Reduced Sensitivity to Experimental Manipulations and Show Less Activity during Chronic Injections/Testing

In the chronic amphetamine study, the genotype differences appeared already prior to injections, as an open field test for baseline activity (7 days before injections started) and a consequent housing in single cages induced the expected decrease in the body weight of WT mice, whereas the body weight of *Negr1*^−/−^ mice stayed stable or even increased slightly during the same time period (Figure 2) resulting in the disappearance of the previously significant body weight difference between genotypes (Supplementary Figure S6). This indicates that *Negr1*^−/−^ mice could be less sensitive to the single-housing stress similar to the phenotype we have previously described in *Lsam*^−/−^ mice [43]. In the baseline open field test, *Negr1*^−/−^ mice spent significantly more time in the center of the field, indicating higher exploratory activity and reduced anxiety (the results of this experiment have been published in [27]. During the course of daily chronic injections, housing in single cages, and testing, however, saline-receiving *Negr1*^−/−^ mice became less active in most of the behavioral parameters that were measured, including total distance traveled and distance in the center (Figure 2). Interestingly, *Negr1*^−/−^ mice performed significantly less rearings both during baseline testing [27] and during the course of chronic testing/saline injections in the current study. Previous studies have found that damage to the hippocampus impairs rearing due to failures in spatial memory, where novelty detection is impaired [65]. The hypothesis that reduced rearing in *Negr1*^−/−^ mice could be the expression of impaired hippocampal morphology is supported by accumulating data on the reduced size of the hippocampus in *Negr1*^−/−^ mice and numerous molecular and cellular alterations in the hippocampi of *Negr1*^−/−^ mice [24,27,30].

### 4.5. Escitalopram-Induced Reduction of 5-HT and DA Turnover Is Enhanced in Negr1^−/−^ Mice

Despite upregulation of the serotonin transporter (Slc6a4), which is the main target of escitalopram in the raphe of Negr1^−/−^ mice, deletion of Negr1 did not induce alterations in the behavior or body weight of mice after chronic administration of escitalopram (Supplementary Figures S7 and S8). However, we found that escitalopram could rescue the significantly smaller volumes of hippocampi that Negr1^−/−^ mice have compared to WT, the phenotype that we have also demonstrated earlier [27]. This result is intriguing, especially as it has been shown earlier that depression-related changes in the hippocampal volume could be prevented by antidepressant treatment [66]. Still, several other studies indicate that hippocampal atrophy persists despite treatment of depression and long-term remission [67]. Interestingly, NEGR1 has been shown to be involved in neurogenesis [21,25,30]. However, it has to be noted that the hippocampal tissue weight, which could be comparable to MRI-based volumetric measures, cannot reflect subtle changes within different layers of the hippocampus (or dorsal vs. ventral region). Therefore, the change in the weight/volume of the hippocampus is more likely to reflect dendritic arborization and not neurogenesis, that only takes place in sub-layers of the dentate gyrus [68].

It is questionable if *Negr1*^−/−^ mice represent an appropriate model for depression despite the strong link of the human *NEGR1* gene with depression phenotypes in accumulating studies because higher levels of NEGR1 have been described in the tissues and body fluids of depressed patients. However, it is likely that NEGR1 is regulating pathways that are linked with depression and, therefore, its role in the reactivity to escitalopram deserves further studies.

Nevertheless, we found clear genotype-specific alterations in biochemical reactions to escitalopram which were evident in the raphe nuclei but not in the hippocampus. Escitalopram induced a decrease in serotonin turnover in both genotypes, but this effect was highly significant only in the raphe of *Negr1*^−/−^ mice. Furthermore, escitalopram induced a robust decrease in dopamine turnover only in *Negr1*^−/−^ mice; this effect was amplified by the increased baseline turnover of dopamine (DOPAC/DA) in the *Negr1*^−/−^ raphe compared to WT. In fact, the significant treatment effect suggests that escitalopram induced the elevation of serotonin in the raphe of both genotypes, but the significant elevation of dopamine was induced only in the raphe of wild-type mice, suggesting that alterations in the dopamine system could affect the serotonergic neurotransmission in *Negr1*^−/−^ raphe. Interestingly, we have previously found that similarly to *Negr1*^−/−^ mice, escitalopram-induced reduction of 5-HT turnover is enhanced in the raphe of *Lsamp*^−/−^ mice [41], indicating overlapping functions of these homologous proteins. In *Negr1*^−/−^ mice, however, we could not see increased 5-HT turnover, which was evident in various brain areas in *Lsamp*^−/−^ mice. At the same time, we detected dopamine-related changes in the raphe of *Negr1*^−/−^ mice that were not seen previously in *Lsamp*^−/−^ mice suggesting distinct functions of these proteins in specific monoaminergic pathways. Accumulating evidence suggests that the serotonergic system has an impact on the activity of dopaminergic neurons in the striatum [69], thus, the altered interplay of dopamine and serotonin might also be responsible for the altered sensitivity to amphetamine in *Negr1*^−/−^ mice, possibly indicated by the increased serotonin metabolite 5-HIAA in the ventral striatum of *Negr1*^−/−^ mice.

### 4.6. Negr1 Deficiency-Induced Alterations in the Monoaminergic Neurotransmission Could Explain Links of NEGR1 with Both Depression and Obesity Phenotypes

Imbalance of monoaminergic neurotransmission in the brain areas, such as mesolimbic pathways [33], raphe [70], and hippocampus have been shown to underlie depressive conditions. Here we show that *Negr1*^−/−^ mice display a time-dependent increase in behavioral sensitization to amphetamine associated with changes in amphetamine-stimulated DA release in the ventral and dorsal striatum, indicating altered reactivity of mesolimbic pathways lacking NEGR1 protein. Mesolimbic pathways underlying reward processing were our special interest as dysfunctional reward processing that has been described both in depressive patients [71,72], and obese subjects [73] could be a shared mechanism underlying both obesity and depression. Anhedonia, one of the core symptoms of depression, has been linked to dysfunctions in the reward system and, in particular, the dopamine system [74]. We have previously shown that *Negr1*^−/−^ mice eat less palatable high-fat food, both if the food consumption was measured for a longer period of time and also during the first 24 h of novel high-fat food exposure [32]. Reduced intake of palatable food in a longer time period is more likely linked with altered metabolic homeostasis in the *Negr1*^−/−^ mice; reduced intake of high-fat food during the first 24 h could be a sign of altered reward processing, which, however, needs further research. Serotonergic signaling contributes to the regulation of both homeostatic and hedonic feeding. The hedonic circuitry reduces reward-related food consumption [75]. Increased serotonin levels found in the hippocampus of *Negr1*^−/−^ mice may contribute to the reduced standard food intake we have previously observed in *Negr1*^−/−^ mice [32] which in turn may explain the lower baseline body weight also observed in the current study. We also showed that *Negr1*^−/−^ mice are less sensitive to stress/injection-induced weight loss, which could reflect the impact of *Negr1* on the body mass index in both mice and humans.

## 5. Conclusions

We show that NEGR1 is expressed in both tyrosine- and tryptophan hydroxylase-positive cells and that *Negr1*^−/−^ mice show altered reactivity to the substances that are targeting monoaminergic neurotransmission. The current study is the first to show alterations in the brain monoaminergic system in mice deficient in *Negr1*. We suggest that these neural circuits could underlay both depressive and obesity-related phenotypes that have been strongly linked with the *NEGR1* gene in human studies.

## 6. Limitations of the Study

Due to technical reasons, the monoamines from dorsal and ventral striatal areas have been measured by using different apparatus and protocols compared to the monoamines measured from hippocampi and raphe. Another limitation of the study was the usage of only male mice. As we have shown in our previous paper [32] that male and female *Negr1*^−/−^ mice show somewhat different metabolic profiles, female mice need to be included in future studies. Heterozygous mice should also be included to specify the gene dose effects in altered phenotypes. Some of the neurodevelopmental and chemical alterations in constitutive knockout mice may not be reversible or may need higher doses/duration of drug treatment. Additionally, important behavioral tests for depressive behavior, such as forced swim tests and glucose preference tests, have not been used in the current study and must be included in future studies focusing on depressive phenotypes in rodents. Studying the monoamine-related biochemistry and pharmacological reactions of mice lacking NEGR1 is only one option for understanding the role of NEGR1 in depression. Accumulating evidence suggests that elevated levels of NEGR1 could be linked with pathological conditions such as obesity and depression, therefore, in future studies, mice with NEGR1 overexpression would serve as a better mouse model for understanding the role of NEGR1 in the pathogenesis of depression and obesity.

## Data Availability

The data will be available upon request from the corresponding author.

## Supplementary Materials

The following supporting information can be downloaded at: https://www.mdpi.com/article/10.3390/brainsci12121696/s1, Figure S1. Amphetamine dose curve and chronic amphetamine experiment; Figure S2. Effects of daily saline injections on activity of Negr1−/− and WT mice; Figure S3. Effects of chronic administration of amphetamine to the levels of dopamine metabolites DOPAC and HVA in the striatal area; Figure S4. Epifluorescent images from immunohistochemical staining of WT mouse hippocampal coronal sections; Figure S5. Expression of Negr1 in the ventral striatum was lower in 129Sv mice compared to Bl6 mice; Figure S6. Effects of chronic administration of amphetamine (3 mg/kg) on the body weight of mice; Figure S7. Effects of chronic administration of escitalopram (10 mg/kg) on the body weight of mice; Figure S8. Escitalopram had no effect on the behavior of the mice; Table S1. The expression level of dopamine system related genes in the VTA; Table S2. The level of dopamine system related genes in the dorsal striatum; Table S3. The expression level of dopamine system related genes in the ventral striatum; Table S4. The level of serotonin system related genes in the raphe; Table S5. The level of dopamine system related genes in the frontal cortex; Table S6. The level of monoamines and their metabolites in VTA of amphetamine experiment mice; Table S7. Values (Mean ± SEM) and statistical parameters for the behavioral analysis of chronic amphetamine experiment; Table S8. The level of monoamines and their metabolites in the dorsal striatum of amphetamine experiment mice; Table S9. The level of monoamines and their metabolites in the ventral striatum of amphetamine experiment mice; Table S10. The level of monoamines and their metabolites in the hippocampus of amphetamine experiment mice; Table S11. The level of monoamines and their metabolites in the hippocampus of escitalopram experiment mice; Table S12. The level of monoamines and their metabolites in the raphe of escitalopram experiment mice; Table S13. Values (Mean ± SEM) and statistical parameters for chronic escitalopram experiment behavioral analysis.
