# Supplementary material for: Depression-Associated Negr1 Gene-Deficiency Induces Alterations in the Monoaminergic Neurotransmission Enhancing Time-Dependent Sensitization to Amphetamine in Male Mice
Source: Brain Sci. 2022 Dec 10;12(12):1696. doi: 10.3390/brainsci12121696 (PMC9776224; doi:10.3390/brainsci12121696)
Supplement: Supplementary file 1 [file brainsci-12-01696-s001.zip › brainsci-2089836-supplementary.pdf]

brain sciences

MDPI

Supplementary Material

Maria Kaare *et al* **(2022) Depression-associated** ***Negr1*** **gene-deficiency induces alterations in the monoaminergic neurotransmission enhancing time-dependent sensitization to amphetamine in male mice**

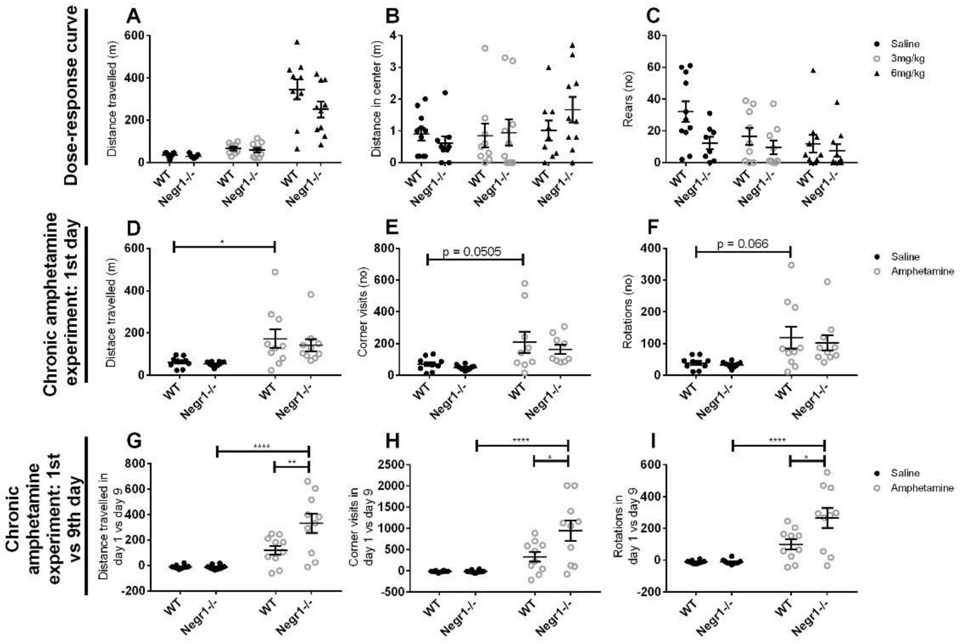

Figure S1. Amphetamine dose curve (A-C) and chronic amphetamine experiment (D-I). All dosage curve mice (cohort Ia) received a single injection of saline or amphetamine on a dosage either 3mg/kg or 6mg/kg. (A) Distance travelled was affected by the genotype ($F_{2,53}$ = 65.31; $p < 0.0001$), while not affected by the treatment ($F_{1,53}$ = 2.881; $p = 0.095$) and genotype x treatment interaction ($F_{2,53}$ = 2.004; p = 0.144), (m), (B) distance in center was not affected by the genotype ($F_{2,52}$ = 1.712; $p = 0.190$), treatment ($F_{1,52}$ = 0.346; $p = 0.558$), and genotype x treatment interaction ($F_{2,52}$ = 1.047; $p = 0.358$), (C) Number of rearings was affected by the treatment ($F_{1,51}$ = 5.899; $p = 0.018$) and genotype ($F_{2,51}$ = 3.098; $p = 0.053$) whereas, not affected by the genotype x treatment interaction ($F_{2,51}$ = 1.259; $p = 0.292$). Figures D-I represent chronic amphetamine experiment mice (cohort Ib). (D-F) The 1st day of amphetamine/saline injection was analyzed to see the acute effect of 3 mg/kg amphetamine on mice. Data shows (D) distance travelled (m), was affected by the treatment ($F_{1,36}$ = 13.94; $p < 0.001$), (E)

number of corner visits, was affected by the treatment ($F_{1,34}$ = 13.08; p = 0.001) and (F) number of rotations, was affected by the treatment ($F_{1,36}$ = 12.47; p = 0.001). (G-I) Days 1 and 9 were compared to see the difference of amphetamine effect between those two days. (G) Distance travelled in day 1 vs day 9, was affected by the genotype ($F_{1,36}$ = 6.32; p = 0.001), treatment $F_{1,36}$ = 33.29; p < 0.0001) and genotype x treatment interaction ($F_{1,36}$ = 6.69; p < 0.05), (H) number of corner visits in day 1 vs day 9 was affected by the genotype ($F_{1,36}$ = 5.31; p = 0.027), treatment ($F_{1,36}$ = 24.1; p < 0.0001) and genotype x treatment interaction ($F_{1,36}$ = 5.31; p < 0.05) and (I) number of rotations in day 1 vs day 9 was affected by the genotype ($F_{1,36}$ = 5.52; p < 0.05), treatment ($F_{1,36}$ = 29.90; p < 0.0001) and genotype x treatment interaction ($F_{1,36}$ = 5.53; p = 0.024). Data represents mean ± SEM, * p < 0.05, **p < 0.01, **** p < 0.0001, two-way ANOVA (Bonferroni *post hoc* test).

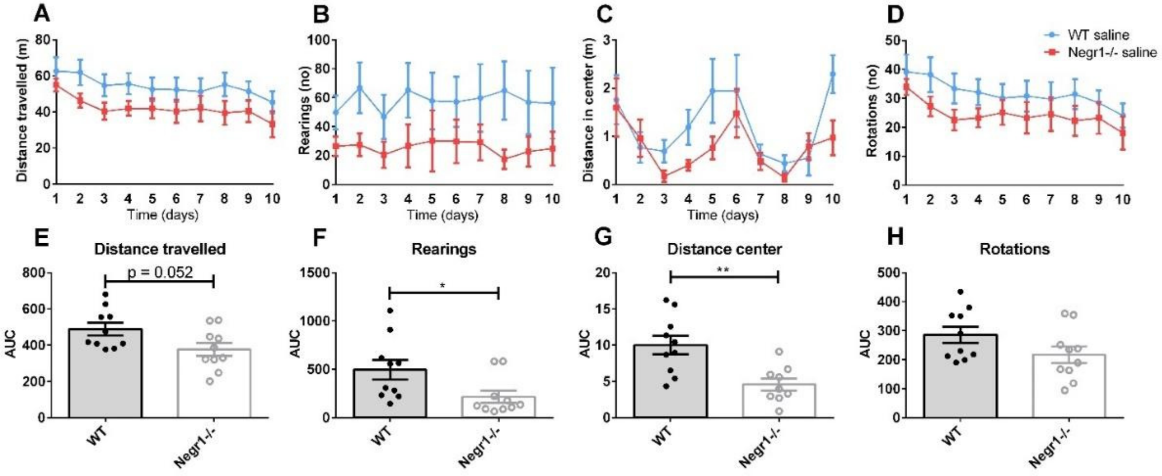

Figure S2. Effects of daily saline injections on activity of *Negr1*$^{-/-}$ and WT mice. The saline-injected groups showed significant effects in the open field test in (A) distance travelled there were time ($F_{9,162}$ = 2.14; < 0.05), genotype ($F_{1,18}$ = 4.91; < 0.05) effect (B) rearings had genotype effect ($F_{1,153}$ = 20.63; p < 0.0001), (C) distance travelled in center had time ($F_{9,162}$ = 3.701; p < 0.001) and genotype effect ($F_{1,18}$ = 5.414; p < 0.05) (D) rotations had time effect ($F_{9,162}$ = 2.072; p < 0.05) (only saline-injected groups from Figure 2A-D are displayed to visualize behavioral differences between genotypes without pharmacological intervention). (E-H) The same parameters quantified as area under the curve (AUC). Data represents mean ± SEM, ++p < 0.01 - genotype effect, *p < 0.05, **p < 0.01 - *post hoc* test, two-way ANOVA (Bonferroni *post hoc* test).

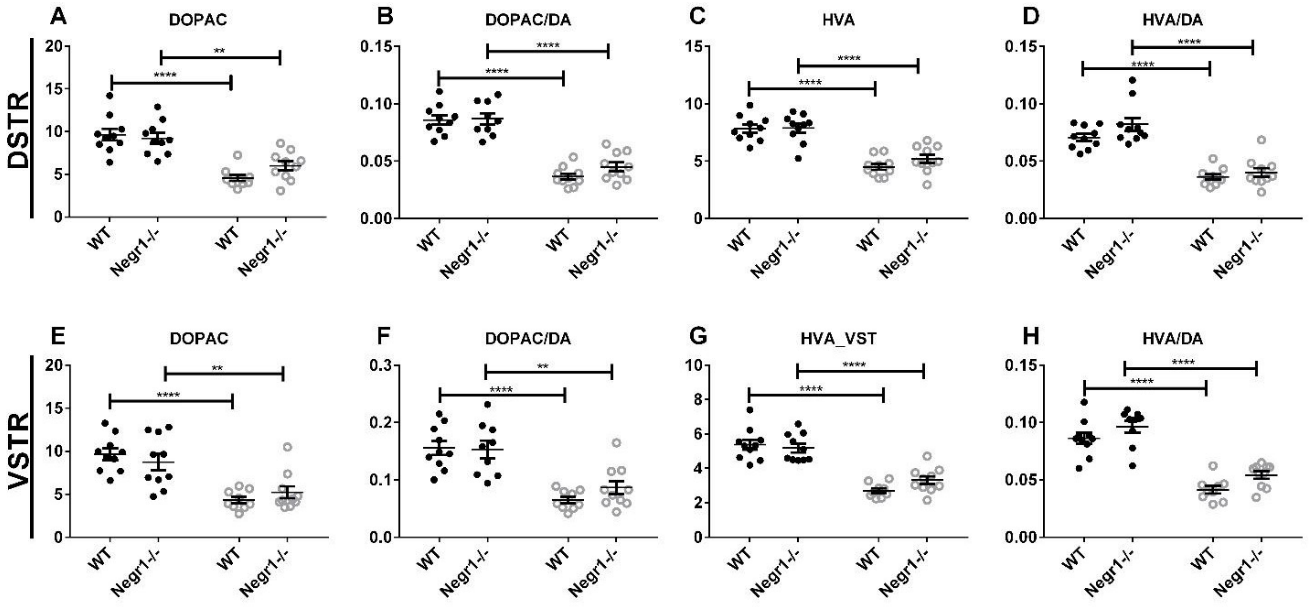

Figure S3. Effects of chronic administration of amphetamine to the levels of dopamine metabolites DOPAC and HVA in the striatal area. The levels of (A) 3,4-Dihydroxyphenylacetic acid (DOPAC), was affected by the treatment ($F_{1,35} = 38.61$; $p < 0.0001$, (B) DOPAC/DA, was affected by the treatment ($F_{1,35} = 45.23$; $p < 0.0001$, (C) Homovanillic acid (HVA), was affected by the treatment ($F_{1,35} = 90.40$; $p < 0.0001$, (D) HVA/DA, was affected by the genotype ($F_{1,34} = 6.870$; $p = 0.013$) and treatment ($F_{1,34} = 100.6$; $p < 0.0001$ in the DSTR. The level of (E) DOPAC, was affected by the treatment ($F_{1,36} = 9.19$; $p = 0.004$), (F) DOPAC/DA, was affected by the treatment ($F_{1,36} = 136.2$; $p < 0.0001$), (G) HVA was affected by the treatment ($F_{1,36} = 5.26$; $p < 0.0001$), (H) HVA/DA was affected by the treatment ($F_{1,36} = 92.09$; $p < 0.0001$) in the VSTR. Data represents mean ± SEM, **$p < 0.01$, **** $p < 0.0001$, two-way ANOVA (Bonferroni *post hoc* test).

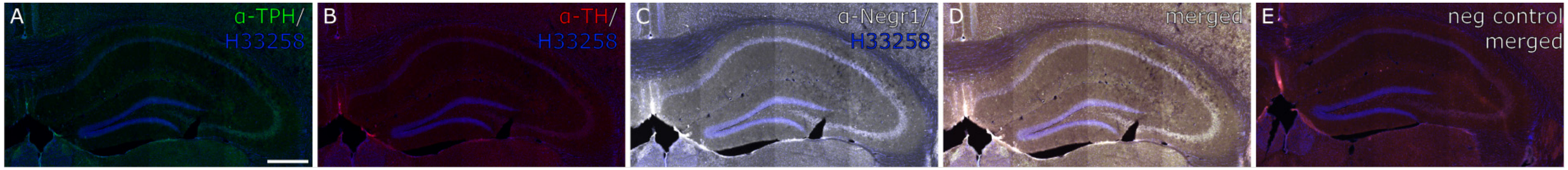

Figure S4. Epifluorescent images from immunohistochemical staining of WT mouse hippocampal coronal sections. Images (A-D) display localization of (B) Tyrosine hydroxylase (TH), (A) Tryptophan hydroxylase 2 (TPH) and (C) NEGR1. (A) In the hippocampus proper we observe diffusely distributed and weakly stained TPH-positive cells. (B) Tyrosine hydroxylase staining is not observable. (C) NEGR1 localization reveals cell bodies throughout the hippocampal formation. With this magnification diffuse signal is observable at the background. (D) merged image from A to C. (E) merged image from negative control displaying the specific binding of the secondary antibodies – weak background staining is observable with Alexa Fluor 647 and Alexa Fluor 594 (single images were obtained using the same acquisition parameters as in images from A to C). Scale bar: 0.5 mm.

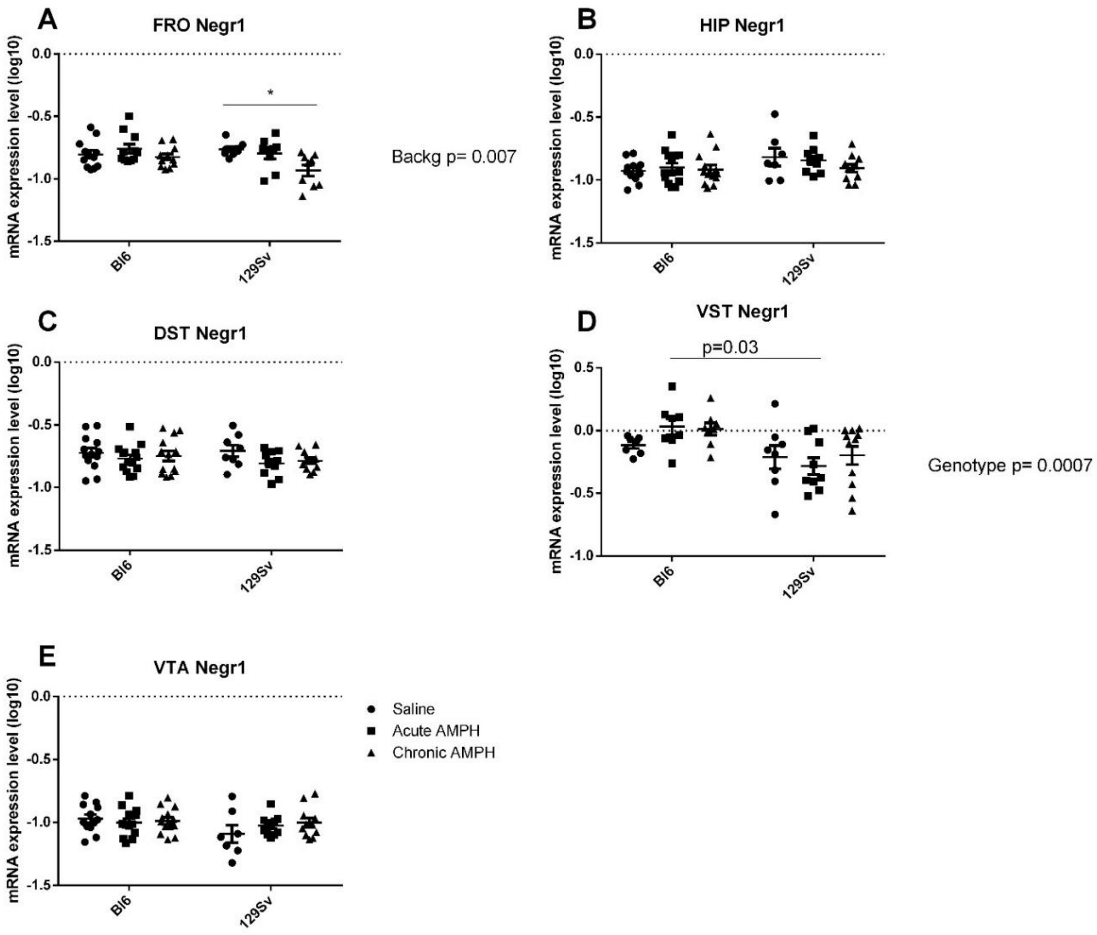

Figure S5. Expression of Negr1 in the ventral striatum was lower in 129Sv mice compared to Bl6 mice. Expression level of *Negr1* in (A) frontal cortex (FRO), (B) hippocampus (HIP), (C) dorsal striatum (DST), (D) ventral striatum (VST) and (E) ventral tegmental area (VTA). Data represents mean ± SEM, *p < 0.05.

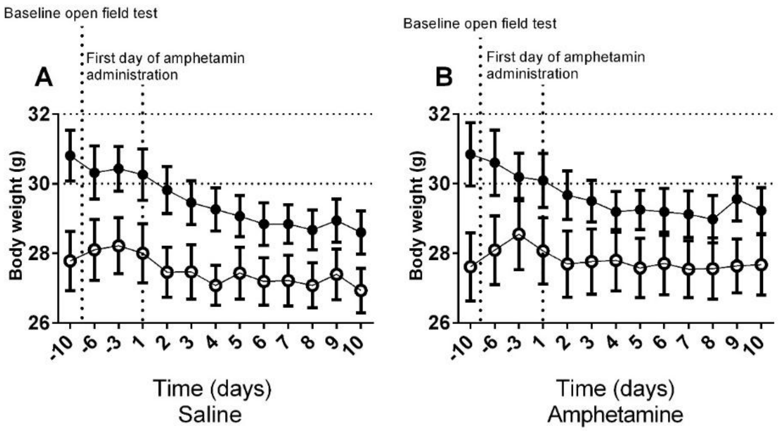

Figure S6. Effects of chronic administration of amphetamine (3 mg/kg) on the body weight of mice. (A) Body weight changes of the saline groups and (B) body weight changes of amphetamine groups. Data represents mean ± SEM.

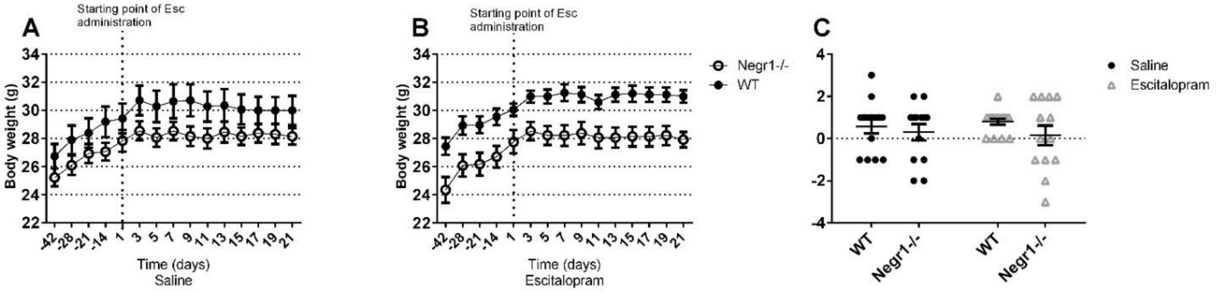

Figure S7. Effects of chronic administration of escitalopram (10 mg/kg) on the body weight of mice. (A) Body weight dynamics of the saline groups and (B) body weight dynamics of escitalopram groups. (C) Body weight changes during the experiment (day 1 vs day 21). Data represents mean ± SEM.

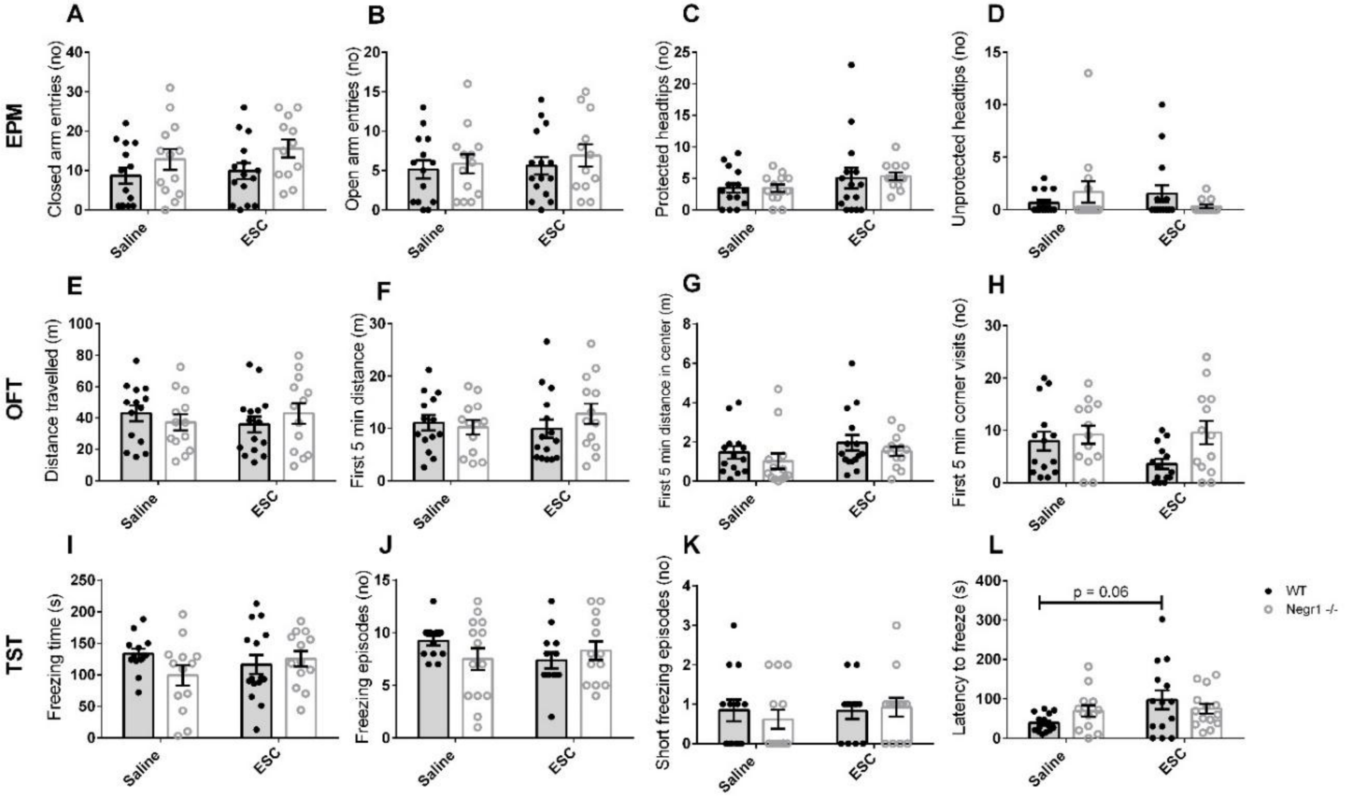

Figure S8. Escitalopram had no effect on the behavior of the mice. Mice received i.p. injection of either saline or 10 mg/kg of escitalopram at a volume of 10 ml/kg for 23 consecutive days. Behavioral changes were evaluated in the (A - D) elevated plus maze (day 16), (E - H) open field test (half of the mice on day 19 and other half of the mice on day 20) and (I - L) tail suspension (day 22) tests. Data represents mean ± SEM.

Table S1. The expression level of dopamine system related genes in the VTA. p values that are statistically significant marked in bold. Data that are not normally distributed marked as red.

			**p value**		**Shapiro-Wilk normality test**	
**VTA**	**WT** Mean ± SEM	***Negr1**$^{-/-}$* Mean ± SEM	**Mann-Whitney U test**	**t test**	**WT** W-value	**Negr1-/-** W-value
Th	0.008 ± 0.002	0.034 ± 0.006	**0.019**	-	0.920	**0.818** **Non-normally distributed**
Drd2	0.021 ± 0.003	0.026 ± 0.003	-	0.252	0.934	0.947
MaoA	0.015 ± 0.004	0.050 ± 0.009	**0.009**	-	**0.812** **Non-normally distributed**	0.907
MaoB	0.016 ± 0.004	0.039 ± 0.005	**0.005**	-	**0.738** **Non-normally distributed**	0.960
Dat	0.015 ± 0.002	0.036 ± 0.007	-	**0.011**	0.866	0.937

Table S2. The level of dopamine system related genes in the dorsal striatum. p values that are statistically significant marked in bold. Data that are not normally distributed marked as red.

			**p value**		**Shapiro-Wilk normality test**	
**Dorsal striatum**	**WT** Mean ± SEM	***Negr1**$^{-/-}$* Mean ± SEM	**Mann-Whitney U test**	**t-test**	**WT** W-value	***Negr1**$^{-/-}$* W-value
Th	0.002 ± 0.0003	0.0007 ± 0.0002	0.128	-	**0.855** **Non-normally distributed**	0.876
Drd1	0.031 ± 0.002	0.029 ± 0.005	-	0.663	0.924	0.958
Drd2	0.113 ± 0.009	0.099 ± 0.012	-	0.346	0.943	0.955
Drd5	0.0008 ± 0.0001	0.0009 ± 0.0001	0.185	-	**0.773** **Non-normally distributed**	0.902
Comt	0.0007 ± 0.00009	0.0006 ± 0.00007	0.483	-	**0.856** **Non-normally distributed**	0.900
MaoA	0.028 ± 0.003	0.021 ± 0.003	0.194	-	0.926	**0.855** **Non-normally distributed**
MaoB	0.016 ± 0.002	0.013 ± 0.002	-	0.225	0.881	0.907

Table S3. The expression level of dopamine system related genes in the ventral striatum. p values that are statistically significant marked in bold. Data that are not normally distributed marked as red.

			P value		Shapiro-Wilk normality test	
**Ventral striatum**	**WT** Mean ± SEM	***Negr1$^{-/-}$*** Mean ± SEM	**Mann-Whitney U test**	**t-test**	**WT** W-value	***Negr1$^{-/-}$*** W-value
Th	0.005 ± 0.002	0.004 ± 0.001	0.962	-	0.826	**0.780** **Non-normally distributed**
Drd1	0.028 ± 0.003	0.03 ± 0.003	-	0.543	0.953	0.949
Drd2	0.055 ± 0.008	0.064 ± 0.008	-	0.401	0.921	0.979
Comt	0.0006 ± 0.0001	0.001 ± 0.0001	**0.014**	-	0.921	**0.979** **Non-normally distributed**
MaoA	0.037 ± 0.004	0.032 ± 0.004	-	0.332	0.904	0.894
MaoB	0.027 ± 0.006	0.021 ± 0.004	0.332	-	0.968	**0.844** **Non-normally distributed**
Dat	0.0003±0.00004	0.0006 ± 0.00008	**0.011**	-	0.912	**0.849** **Non-normally distributed**

Table S4. The level of serotonin system related genes in the raphe. p values that are statistically significant marked in bold. Data that are not normally distributed marked as red.

			**P value**		**Shapiro-Wilk normality test**	
**Raphe**	**WT**<br>Mean ± SEM	***Negr1$^{-/-}$***<br>Mean ± SEM	**Mann-Whitney U test**	**t-test**	**WT**<br>W-value	***Negr1$^{-/-}$***<br>W-value
Slc6a4	0.004 ± 0.0005	0.006 ± 0.0007	-	**0.009**	0.947	0.924
MaoA	0.051 ± 0.007	0.045 ± 0.005	-	0.521	0.926	0.986
MaoB	0.029 ± 0.004	0.026 ± 0.003	-	0.662	0.914	0.939
Tph2	0.015 ± 0.003	0.020 ± 0.004	0.671	-	0.896	**0.855**<br>**Non-normally distributed**

Table S5. The level of dopamine system related genes in the frontal cortex. Data that are not normally distributed marked as red.

			p value		Shapiro-Wilk normality test	
**FRO**	**WT** Mean ± SEM	***Negr1-/-*** Mean ± SEM	**Mann-Whitney U test**	**t test**	**WT** W-value	**Negr1-/-** W-value
Th	0.0009 ± 0.0002	0.002 ± 0.0005	0.469	-	0.942	**0.767** Non-normally distributed
Drd1	0.002 ± 0.0003	0.002 ± 0.0003	0.380	-	0.877	**0.691** Non-normally distributed
Drd2	0.001 ± 0.0003	0.001 ± 0.0001	-	0.386	0.907	0.915
Drd5	0.001 ± 0.0002	0.001 ± 0.0002	0.402	-	0.941	**0.737** Non-normally distributed
Comt	0.002 ± 0.0002	0.002 ± 0.0004	-	0.183	0.930	0.929
MaoA	0.036 ± 0.003	0.040 ± 0.002	-	0.296	0.894	0.921
MaoB	0.034 ± 0.007	0.037 ± 0.007	0.643	-	0.884	**0.687** Non-normally distributed
Dat	0.0005 ± 7.90e-005	0.0006 ± 0.0001	-	0.854	0.918	0.855

Table S6. The level of monoamines and their metabolites in VTA of amphetamine experiment mice. p values that are statistically significant marked in bold.

	**Saline**		**Amphetamine**		**Two-way ANOVA**		
**VTA**	**WT**<br>Mean ± SEM	***Negr1*** $^{-/-}$<br>Mean ± SEM	**WT**<br>Mean ± SEM	***Negr1*** $^{-/-}$<br>Mean ± SEM		**F**	**p**
Th	0.030 ± 0.003	0.43 ± 0.007	0.065 ± 0.014	0.032 ± 0.005	**Treatment**<br>**Genotype**<br>**Treatment X genotype**	2.08<br>1.35<br>8.30	0.158<br>0.253<br>**0.007**
Drd2	0.016 ± 0.0008	0.018 ± 0.0012	0.019 ± 0.002	0.015 ± 0.001	**Treatment**<br>**Genotype**<br>**Treatment X genotype**	0.06<br>0.22<br>6.09	0.807<br>0.640<br>**0.019**
Comt	0.001 ± 0.0001	0.002 ± 0.0002	0.002 ± 0.0003	0.001 ± 0.0001	**Treatment**<br>**Genotype**<br>**Treatment X genotype**	0.31<br>0.21<br>5.15	0.584<br>0.652<br>**0.031**
MaoA	0.088 ± 0.004	0.092 ± 0.005	0.091 ± 0.006	0.086 ± 0.007	**Treatment**<br>**Genotype**	0.11<br>0.01	0.748

					**Treatment X genotype**	0.65	0.920<br>0.426
MaoB	0.042 ± 0.002	0.047 ± 0.003	0.046 ± 0.003	0.046 ± 0.004	**Treatment**<br>**Genotype**<br>**Treatment X genotype**	0.11<br>0.59<br>0.63	0.741<br>0.449<br>0.432
Dat	0.049 ± 0.004	0.060 ± 0.004	0.068 ± 0.010	0.050 ± 0.003	**Treatment**<br>**Genotype**<br>**Treatment X genotype**	0.55<br>0.38<br>5.59	0.462<br>0.543<br>**0.024**

Table S7. Values (Mean ± SEM) and statistical parameters for the behavioral analysis of chronic amphetamine experiment. p values that are statistically significant marked in bold.

	WT	***Negr1$^{-/-}$***	WT	***Negr1$^{-/-}$***	**Repeated measures 3way ANOVA**		
	Saline<br>Mean ± SEM		Amphetamine<br>Mean ± SEM			**F**	**p**
**Distance travelled**							

Day 1	62.72 ± 7.72	54.88 ± 3.55	172.41 ± 43.85	142.96 ± 28.49	**Time**<br>**Treatment**<br>**Genotype**<br>**Time x treatment**<br>**Time x genotype**<br>**Treatment x genotype**<br>**Time x treatment x genotype**	5.65<br>67.24<br>14.03<br>7.01<br>0.68<br>22.84<br>0.67	**0.001**<br>**<0.0001**<br>**0.001**<br>**<0.0001**<br>0.517<br>**<0.001**<br>0.729
Day 2	61.85 ± 7.03	46.32 ± 3.96	172.06 ± 47.29	186.69 ± 53.12			
Day 3	54.73 ± 6.02	40.37 ± 4.73	221.53 ± 55.46	255.14 ± 62.14			
Day 4	55.66 ± 5.85	41.92 ± 4.03	226.73 ± 56.25	302.80 ± 66.37			
Day 5	52.58 ± 6.51	41.94 ± 5.51	213.12 ± 48.42	338.09 ± 69.94			
Day 6	52.34 ± 6.60	40.42 ± 6.39	237.52 ± 59.54	380.03 ± 66.62			
Day 7	51.29 ± 7.42	41.92 ± 7.06	273.00 ± 57.14	439.55 ± 76.71			
Day 8	55.18 ± 6.53	39.48 ± 6.56	287.13 ± 60.61	434.31 ± 72.12			
Day 9	51.40 ± 5.56	40.51 ± 6.15	292.30 ± 49.68	473.16 ± 78.60			
Day 10	45.34 ± 6.07	33.26 ± 7.24	305.68 ± 58.74	455.84 ± 75.01			
**Corner visits**							
Day 1	70.10 ± 12.87	47.90 ± 5.02	312.50 ± 119.27	242.60 ± 83.64	**Time**<br>**Treatment**	5.06<br>63.32	**0.002**
Day 2	74.40 ± 13.95	39.70 ± 5.63	318.00 ± 134.64	384.50 ± 156.88			

Day 3	60.80 ± 11.83	35.30 ± 8.99	472.10 ± 161.64	551.30 ± 173.05	**Genotype**<br>**Time x treatment**<br>**Time x genotype**<br>**Treatment x genotype**<br>**Time x treatment x genotype**	12.02<br>5.54<br>0.53<br>17.36<br>0.51	**<0.0001**<br>**0.002**<br>**<0.0001**<br>0.595<br>**<0.001**<br>0.861
Day 4	64.10 ± 11.56	38.00 ± 4.84	465.00 ± 163.02	650.20 ± 195.04			
Day 5	57.60 ±12.37	40.00 ± 8.35	409.70 ± 144.72	761.00 ± 210.55			
Day 6	61.20 ± 11.53	34.90 ± 9.32	530.50 ± 171.22	899.10 ± 205.68			
Day 7	59.10 ± 13.63	35.40 ± 8.88	634.90 ± 172.69	1107.90 ± 239.22			
Day 8	64.60 ± 13.22	30.40 ± 6.67	668.20 ± 183.05	1036.00 ± 218.75			
Day 9	55.60 ± 10.41	33.40 ± 7.36	645.20 ± 154.14	1189.90 ± 260.74			
Day 10	47.80 ± 10.96	27.70 ± 9.05	723.20 ± 177.82	1115.70 ± 239.06			
**Rotations**							
Day 1	39.17 ± 5.98	33.99 ± 2.77	118.99 ± 34.32	102.77 ± 23.47	**Time**<br>**Treatment**<br>**Genotype**<br>**Time x treatment**<br>**Time x genotype**	5.48<br>43.95<br>15.12<br>6.81<br>0.77	**0.001**<br>**<0.0001**<br>**0.001**<br>**<0.0001**
Day 2	38.22 ± 6.02	27.26 ± 3.37	121.96 ± 38.65	135.43 ± 43.51			
Day 3	33.40 ± 5.21	22.51 ± 3.58	157.31 ± 45.51	192.48 ± 52.38			
Day 4	32.10 ± 4.51	23.31 ± 3.35	163.93 ± 47.31	237.93 ± 57.10			

Day 5	30.08 ± 4.92	25.11 ± 4.31	153.64 ± 42.82	259.07 ± 57.82	**Treatment x genotype**<br>**Time x treatment x genotype**	21.03<br>0.72	0.476<br>**<0.001**<br>0.687
Day 6	30.80 ± 5.32	23.29 ± 5.41	171.48 ± 49.18	293.53 ± 56.97			
Day 7	29.73 ± 5.92	24.52 ± 5.85	196.66 ± 48.44	341.96 ± 65.17			
Day 8	31.46 ± 5.20	22.27 ± 5.19	211.02 ± 51.90	346.32 ± 65.83			
Day 9	28.42 ± 4.37	23.17 ± 5.46	218.47 ± 44.41	368.55 ± 67.79			
Day 10	24.06 ± 4.25	18.00 ± 5.68	225.76 ± 50.24	361.44 ± 63.93			

Table S8. The level of monoamines and their metabolites in the dorsal striatum of amphetamine experiment mice. p values that are statistically significant marked in bold.

	**Saline**		**Amphetamine**		**Two-way ANOVA**		
**Dorsal striatum**	**WT**<br>Mean ± SEM	***Negr1$^{-/-}$***<br>Mean ± SEM	**WT**<br>Mean ± SEM	***Negr1$^{-/-}$***<br>Mean ± SEM		**F**	**p**
DA	111.99 ± 5.45	101.02 ± 8.62	126.15 ± 6.12	135.70 ± 10.89	**Treatment**<br>**Genotype**<br>**Treatment X genotype**	9.19<br>0.008<br>1.62	**0.005**<br>0.930<br>0.211
DOPAC	9.64 ± 0.69	9.21 ± 0.62	4.57 ± 0.35	5.99 ± 0.54	**Treatment**<br>**Genotype**	54.63<br>0.78<br>2.69	**<0.0001**<br>0.383<br>0.110

					**Treatment X genotype**		
HVA	7.84 ± 0.36	7.89 ± 0.40	4.52 ± 0.27	5.20 ± 0.37	**Treatment**<br>**Genotype**<br>**Treatment X genotype**	75.26<br>1.10<br>0.81	**<0.0001**<br>0.302<br>0.375
3-MT	6.21 ± 0.46	6.71 ± 0.46	7.56 ± 0.56	8.50 ± 0.40	**Treatment**<br>**Genotype**<br>**Treatment X genotype**	11.21<br>2.41<br>0.22	**0.002**<br>0.129<br>0.642
DOPAC/DA	0.09 ± 0.005	0.10 ± 0.02	0.04 ± 0.003	0.05 ± 0.004	**Treatment**<br>**Genotype**<br>**Treatment X genotype**	136.2<br>1.53<br>0.96	**<0.0001**<br>0.224<br>0.335
HVA/DA	0.07 ± 0.004	0.08 ± 0.006	0.04 ± 0.003	0.04 ± 0.004	**Treatment**<br>**Genotype**<br>**Treatment X genotype**	92.09<br>3.70<br>0.91	**<0.0001**<br>0.062<br>0.346
(DOPAC+HVA)/DA	0.16 ± 0.006	0.18 ± 0.02	0.07 ± 0.004	0.09 ± 0.01	**Treatment**<br>**Genotype**<br>**Treatment X genotype**	187.0<br>3.01<br>0.13	**<0.0001**<br>0.092<br>0.720
3-MT/DA	0.06 ± 0.004	0.07 ± 0.005	0.06 ± 0.004	0.07 ± 0.005	**Treatment**<br>**Genotype**	0.005<br>5.30	0.947<br>**0.027**

					**Treatment X genotype**	1.06	0.311
NA	4.10 ± 0.85	5.77 ± 0.52	3.29 ± 0.55	4.94 ± 0.95	**Treatment**<br>**Genotype**<br>**Treatment X genotype**	3.50<br>3.91<br>0.38	0.070<br>0.056<br>0.539
NMN	9.79 ± 0.25	10.92 ± 0.74	9.12 ± 0.65	10.46 ± 0.59	**Treatment**<br>**Genotype**<br>**Treatment X genotype**	0.92<br>4.46<br>0.03	0.344<br>**0.042**<br>0.858
NMN/NA	3.30 ± 0.92	2.05 ± 0.22	3.44 ± 0.62	3.07 ± 0.73	**Treatment**<br>**Genotype**<br>**Treatment X genotype**	0.78<br>1.54<br>0.46	0.382<br>0.223<br>0.503
5-HT	10.41 ± 0.87	10.45 ± 0.10	10.99 ± 0.91	11.79 ± 0.86	**Treatment**<br>**Genotype**<br>**Treatment X genotype**	1.13<br>0.21<br>0.18	0.294<br>0.647<br>0.678
5-HIAA	3.17 ± 0.11	3.01 ± 0.18	2.76 ± 0.20	3.19 ± 0.34	**Treatment**<br>**Genotype**<br>**Treatment X genotype**	0.26<br>0.36<br>1.84	0.615<br>0.552<br>0.183
5-HIAA/5-HT	0.32 ± 0.02	0.31 ± 0.03	0.26 ± 0.03	0.27 ± 0.03	**Treatment**<br>**Genotype**	3.322<br>0.005<br>0.08	0.077<br>0.943<br>0.782

					**Treatment X genotype**		

Table S9. The level of monoamines and their metabolites in the ventral striatum of amphetamine experiment mice. p values that are statistically significant marked in bold.

	Saline		Amphetamine		Two-way ANOVA		
**Ventral striatum**	**WT** Mean ± SEM	***Negr1$^{-/-}$*** Mean ± SEM	**WT** Mean ± SEM	***Negr1$^{-/-}$*** Mean ± SEM		**F**	**p**
DA	58.84 ± 3.58	52.50 ± 4.63	68.70 ± 5.74	63.51 ± 6.10	**Treatment**<br>**Genotype**<br>**Treatment X genotype**	4.09<br>1.25<br>0.01	0.051<br>0.271<br>0.911
DOPAC	9.65 ± 0.66	8.75 ± 0.96	4.32 ± 0.37	5.22 ± 0.69	**Treatment**<br>**Genotype**<br>**Treatment X genotype**	45.23<br>0.66<br>1.02	**<0.0001**<br>0.422<br>0.319
HVA	5.38 ± 0.29	5.19 ± 0.25	2.71 ± 0.14	3.31 ± 0.22	**Treatment**<br>**Genotype**<br>**Treatment X genotype**	90.40<br>0.73<br>2.77	**<0.0001**<br>0.400<br>0.105
3-MT	4.69 ± 0.43	4.93 ± 0.26	6.57 ± 0.96	6.49 ± 0.54	**Treatment**	9.77	**0.004**

					**Genotype**<br>**Treatment X genotype**	1.82<br>0.57	0.186<br>0.456
DOPAC/DA	0.16 ± 0.02	0.18 ± 0.03	0.07 ± 0.01	0.09 ± 0.01	**Treatment**<br>**Genotype**<br>**Treatment X genotype**	45.23<br>0.66<br>1.02	**<0.0001**<br>0.422<br>0.319
HVA/DA	0.09 ± 0.005	0.11 ± 0.01	0.04 ± 0.003	0.05 ± 0.003	**Treatment**<br>**Genotype**<br>**Treatment X genotype**	100.6<br>6.87<br>0.09	**<0.0001**<br>**0.013**<br>0.760
(DOPAC+HVA)/DA	0.25 ± 0.02	0.29 ± 0.04	0.11 ± 0.01	0.14 ± 0.01	**Treatment**<br>**Genotype**<br>**Treatment X genotype**	69.99<br>2.05<br>0.81	**<0.0001**<br>0.162<br>0.374
3-MT/DA	0.08 ± 0.01	0.10 ± 0.01	0.11 ± 0.04	0.10 ± 0.05	**Treatment**<br>**Genotype**<br>**Treatment X genotype**	0.40<br>12.51<br>1.043e-007	0.530<br>**0.001**<br>1.000
NA	5.54 ± 0.61	6.43 ± 0.84	4.07 ± 0.49	5.30 ± 0.52	**Treatment**<br>**Genotype**<br>**Treatment X genotype**	4.22<br>2.81<br>0.07	**0.048**<br>0.103<br>0.189
NMN	7.50 ± 0.39	8.06 ± 0.47	6.13 ± 0.27	7.31 ± 0.43	**Treatment**<br>**Genotype**	7.05<br>4.75	**0.012**<br>**0.036**

					**Treatment X genotype**	0.60	0.446
NMN/NA	1.56 ± 0.23	1.52 ± 0.25	1.79 ± 0.32	1.51 ± 0.19	**Treatment**<br>**Genotype**<br>**Treatment X genotype**	0.21<br>0.43<br>0.23	0.649<br>0.517<br>0.636
5-HT	17.11 ± 3.64	16.90 ± 2.97	16.36 ± 2.89	16.64 ± 2.56	**Treatment**<br>**Genotype**<br>**Treatment X genotype**	0.03<br>8.361e-005<br>0.006	0.870<br>0.993<br>0.936
5-HIAA	2.63 ± 0.13	3.48 ± 0.42	1.98 ± 0.15	2.62 ± 0.19	**Treatment**<br>**Genotype**<br>**Treatment X genotype**	10.67<br>10.06<br>0.18	**0.003**<br>**0.003**<br>0.674
5-HIAA/5-HT	0.19 ± 0.03	0.23 ± 0.03	0.14 ± 0.02	0.18 ± 0.02	**Treatment**<br>**Genotype**<br>**Treatment X genotype**	3.53<br>1.95<br>0.23	0.069<br>0.172<br>0.631

Table S10. The level of monoamines and their metabolites in the hippocampus of amphetamine experiment mice. p values that are statistically significant marked in bold.

	**Saline**		**Amphetamine**		**Two-way ANOVA**		
**Hippocampus**	**WT** Mean ± SEM	***Negr1$^{-/-}$*** Mean ± SEM	**WT** Mean ± SEM	***Negr1$^{-/-}$*** Mean ± SEM		**F**	**p**
DA	0.21 ± 0.04	0.29 ± 0.04	0.32 ± 0.03	0.40 ± 0.10	**Treatment**	1.34	0.255
					**Genotype**	0.05	0.834
					**Treatment X genotype**	3.68	0.064
3-MT	4.76 ± 1.22	7.67 ± 1.40	3.72 ± 1.02	7.72 ± 1.84	**Treatment**	0.12	0.728
					**Genotype**	6.00	**0.019**
					**Treatment X genotype**	0.15	0.701
3-MT/DA	24.14 ± 5.70	0.26 ± 11.08	12.83 ± 3.80	28.89 ± 9.50	**Treatment**	1.26	0.269
					**Genotype**	2.91	0.097
					**Treatment X genotype**	0.07	0.797
5-HT	2.13 ± 0.35	3.25 ± 0.35	4.63 ± 0.53	4.76 ± 0.62	**Treatment**	17.51	**<0.001**
					**Genotype**	1.70	0.201
					**Treatment X genotype**	1.08	0.306

5-HIAA	2.88 ± 0.92	3.56 ± 0.71	2.37 ± 0.59	1.38 ± 0.37	**Treatment**<br>**Genotype**<br>**Treatment X genotype**	4.18<br>0.05<br>1.62	**0.049**<br>0.822<br>0.212
5-HIAA/5-HT	1.70 ± 0.54	1.30 ± 0.34	0.61 ± 0.20	0.33 ± 0.09	**Treatment**<br>**Genotype**<br>**Treatment X genotype**	7.32<br>0.17<br>1.18	**0.011**<br>0.682<br>0.286
Trp	0.86 ± 0.16	0.82 ± 0.03	0.76 ± 0.07	0.95 ± 0.20	**Treatment**<br>**Genotype**<br>**Treatment X genotype**	0.002<br>0.56<br>0.22	0.961<br>0.458<br>0.641
Tyr	373.14 ± 28.56	448.25 ± 24.46	334.73 ± 26.05	382.42 ± 47.38	**Treatment**<br>**Genotype**<br>**Treatment X genotype**	7.78<br>2.41<br>1.74	**0.009**<br>0.130<br>0.196
NA	1.26 ± 0.13	1.68 ± 0.11	0.56 ± 0.10	1.54 ± 0.16	**Treatment**<br>**Genotype**<br>**Treatment X genotype**	0.01<br>4.36<br>1.52	0.912<br>**0.044**<br>0.225
NMN	0.52 ± 0.24	0.44 ± 0.04	0.56 ± 0.04	1.19 ± 0.61	**Treatment**<br>**Genotype**<br>**Treatment X genotype**	19.44<br>3.71<br>2.16	**<0.001**<br>0.063<br>0.152

MN	0.29 ± 0.04	0.34 ± 0.04	0.31 ± 0.10	0.31 ± 0.04	**Treatment**<br>**Genotype**<br>**Treatment X genotype**	0.008<br>0.56<br>0.46	0.930<br>0.459<br>0.504
NMN/NA	0.24 ± 0.02	0.26 ± 0.02	0.40 ± 0.04	0.41 ± 0.04	**Treatment**<br>**Genotype**<br>**Treatment X genotype**	28.47<br>0.18<br>0.06	**<0.0001**<br>0.672<br>0.814
Tyramine	0.04 ± 0.004	0.05 ± 0.005	0.05 ± 0.002	0.04 ± 0.004	**Treatment**<br>**Genotype**<br>**Treatment X genotype**	0.13<br>0.30<br>5.82	0.717<br>0.586<br>**0.021**

Table S11. The level of monoamines and their metabolites in the hippocampus of escitalopram experiment mice. p values that are statistically significant marked in bold.

	**Saline**		**Escitalopram**		**Two-way ANOVA**		
**Hippocampus**	**WT**<br>Mean ± SEM	***Negr1$^{-/-}$***<br>Mean ± SEM	**WT**<br>Mean ± SEM	***Negr1$^{-/-}$***<br>Mean ± SEM		**F**	**p**
DA	0.12 ± 0.02	0.14 ± 0.02	0.18 ± 0.04	0.18 ± 0.05	**Treatment**<br>**Genotype**<br>**Treatment X genotype**	1.28<br>0.29<br>1.56	0.246<br>0.591<br>0.218
3-MT	7.55 ± 1.21	9.94 ± 1.87	16.17 ± 4.10	20.90 ± 3.72	**Treatment**<br>**Genotype**	10.36<br>1.37	**0.002**<br>0.247

					**Treatment X genotype**	0.15	0.702
3-MT/DA	79.63 ± 15.78	82.79 ± 8.42	178.91 ± 63.4	217.10± 72.46	**Treatment**	1.41	0.242
					**Genotype**	3.58	0.064
					**Treatment X genotype**	3.10	0.085
5-HT	2.70 ± 0.34	3.59 ± 0.34	2.74 ± 0.31	3.37 ± 0.34	**Treatment**	0.07	0.791
					**Genotype**	5.40	**0.024**
					**Treatment X genotype**	0.15	0.696
5-HIAA	2.07 ± 0.55	3.95 ± 1.20	2.06 ± 0.46	3.84 ± 0.75	**Treatment**	2.02	0.162
					**Genotype**	8.64	**0.005**
					**Treatment X genotype**	0.26	0.616
5-HIAA/5-HT	2.64 ± 1.92	1.31 ± 0.46	0.85 ± 0.18	1.23 ± 0.25	**Treatment**	1.39	0.245
					**Genotype**	1.88	0.177
					**Treatment X genotype**	0.36	0.554
Trp	0.68 ± 0.10	1.03 ± 0.26	0.77 ± 0.09	0.98 ± 0.19	**Treatment**	0.49	0.489
					**Genotype**	0.65	0.423
					**Treatment X genotype**	0.12	0.733
Tyr	380.48 ± 29.58	499.77 ± 40.46	402.29 ± 37.10	421.02 ± 22.68	**Treatment**	0.72	0.399
					**Genotype**	4.25	**0.045**
					**Treatment X genotype**	2.25	0.140
NA	1.48 ± 0.12	1.60 ± 0.19	1.39 ± 0.13	1.67 ± 0.13	**Treatment**	0.30	0.588
					**Genotype**	4.14	**0.047**
					**Treatment X genotype**	0.03	0.874
NMN	0.40 ± 0.05	1.02 ± 0.42	0.36 ± 0.03	0.45 ± 0.06	**Treatment**	4.57	**0.038**

					**Genotype**<br>**Treatment X genotype**	10.55<br>1.25	**0.002**<br>0.270
MN	0.42 ± 0.06	0.44 ± 0.05	0.35 ± 0.04	0.50 ± 0.13	**Treatment**<br>**Genotype**<br>**Treatment X genotype**	0.009<br>3.38<br>1.67	0.923<br>0.072<br>0.202
NMN/NA	0.28 ± 0.03	0.38 ± 0.05	0.28 ± 0.03	0.28 ± 0.04	**Treatment**<br>**Genotype**<br>**Treatment X genotype**	2.03<br>1.99<br>1.98	0.161<br>0.164<br>0.166
Tyramine	0.04 ± 0.004	0.05 ± 0.01	0.05 ± 0.004	0.06 ± 0.004	**Treatment**<br>**Genotype**<br>**Treatment X genotype**	0.31<br>5.93<br>0.0007	0.579<br>**0.018**<br>0.979

Table S12. The level of monoamines and their metabolites in the raphe of escitalopram experiment mice. p values that are statistically significant marked in bold.

	**Saline**		**Escitalopram**		**Two-way ANOVA**		
**Raphe**	**WT**<br>Mean ± SEM	***Negr1$^{-/-}$***<br>Mean ± SEM	**WT**<br>Mean ± SEM	***Negr1$^{-/-}$***<br>Mean ± SEM		**F**	**p**
5-HT	2.78 ± 0.23	2.79 ± 0.31	3.58 ± 0.34	3.38 ± 0.43	**Treatment**<br>**Genotype**<br>**Treatment X genotype**	6.36<br>0.02<br>0.01	**0.015**<br>0.892<br>0.906
5-HIAA	3.55 ± 0.32	4.23 ± 0.31	3.42 ± 0.15	4.08 ± 0.64	**Treatment**	6.23	**0.016**

					**Genotype**	0.38	0.542
					**Treatment X genotype**	1.04	0.313
5-HIAA/5-HT	1.35 ± 0.16	1.75 ± 0.23	1.13 ± 0.18	1.35 ± 0.24	**Treatment**	19.11	**<0.0001**
					**Genotype**	0.71	0.405
					**Treatment X genotype**	1.38	0.246
Trp	21.33 ± 1.72	26.22 ± 2.14	23.56 ± 2.04	22.06 ± 1.64	**Treatment**	0.26	0.614
					**Genotype**	0.80	0.377
					**Treatment X genotype**	2.84	0.098
Tyr	343.85 ± 19.82	367.47 ± 27.41	342.60 ± 21.36	358.81 ± 31.15	**Treatment**	0.04	0.844
					**Genotype**	0.64	0.429
					**Treatment X genotype**	0.02	0.883
Adrenaline	1.39 ± 0.08	1.50 ± 0.11	1.40 ± 0.10	1.45 ± 0.14	**Treatment**	0.03	0.870
					**Genotype**	0.56	0.457
					**Treatment X genotype**	0.10	0.748
NA	2.12 ± 0.12	2.48 ± 0.33	2.24 ± 0.18	1.94 ± 0.16	**Treatment**	0.16	0.691
					**Genotype**	0.50	0.483
					**Treatment X genotype**	1.37	0.247
NMN	1.22 ± 0.25	1.11 ± 0.10	0.95 ± 0.06	0.96 ± 0.07	**Treatment**	1.72	0.195
					**Genotype**	1.15	0.288

					**Treatment X genotype**	0.83	0.368
MN	1.85 ± 0.27	2.25 ± 0.33	1.65 ± 0.21	1.60 ± 0.26	**Treatment**	2.62	0.112
					**Genotype**	0.48	0.490
					**Treatment X genotype**	0.75	0.392
MN/A	1.34 ± 0.18	1.58 ± 0.23	1.25 ± 0.18	1.20 ± 0.21	**Treatment**	1.43	0.238
					**Genotype**	0.26	0.614
					**Treatment X genotype**	0.53	0.471
NMN/NA	0.60 ± 0.13	0.51 ± 0.07	0.45 ± 0.04	0.52 ± 0.06	**Treatment**	0.01	0.905
					**Genotype**	0.94	0.337
					**Treatment X genotype**	0.23	0.632
DA	0.23 ± 0.02	0.22 ± 0.02	0.25 ± 0.02	0.22 ± 0.03	**Treatment**	3.19	0.081
					**Genotype**	3.08	0.086
					**Treatment X genotype**	4.11	**0.048**
DOPAC	0.37 ± 0.03	0.48 ± 0.05	0.38 ± 0.04	0.32 ± 0.05	**Treatment**	3.79	0.057
					**Genotype**	0.56	0.459
					**Treatment X genotype**	5.80	**0.020**
HVA	0.26 ± 0.05	0.37 ± 0.07	0.25 ± 0.03	0.24 ± 0.06	**Treatment**	3.50	0.068
					**Genotype**	0.13	0.725
						2.38	0.130

					**Treatment X genotype**		
3-MT	0.56 ± 0.05	0.70 ± 0.09	0.61 ± 0.05	0.54 ± 0.07	**Treatment**<br>**Genotype**<br>**Treatment X genotype**	0.77<br>0.32<br>2.65	0.385<br>0.573<br>0.110
DOPAC/DA	1.66 ± 0.16	2.14 ± 0.11	1.59 ± 0.17	1.50 ± 0.18	**Treatment**<br>**Genotype**<br>**Treatment X genotype**	17.53<br>2.51<br>6.03	**<0.001**<br>0.120<br>**0.018**
HVA/DA	1.24 ± 0.23	1.63 ± 0.28	1.07 ± 0.14	1.10 ± 0.25	**Treatment**<br>**Genotype**<br>**Treatment X genotype**	2.76<br>0.16<br>2.18	0.103<br>0.687<br>0.146
(DOPAC+HVA)/DA	2.90 ± 0.34	3.77 ± 0.30	2.65 ± 0.29	2.60 ± 0.42	**Treatment**<br>**Genotype**<br>**Treatment X genotype**	4.51<br>1.52<br>1.93	**0.039**<br>0.223<br>0.171
Tyramine	0.04 ± 0.006	0.08 ± 0.02	0.04 ± 0.006	0.06 ± 0.02	**Treatment**<br>**Genotype**<br>**Treatment X genotype**	2.29<br>5.38<br>2.82	0.137<br>**0.025**<br>0.100

Table S13. Values (Mean ± SEM) and statistical parameters for chronic escitalopram experiment behavioral analysis. p values that are statistically significant marked in bold.

	WT	***Negr1***$^{-/-}$	WT	***Negr1***$^{-/-}$	**Two-way ANOVA**		
	Saline<br>Mean ± SEM		Escitalopram<br>Mean ± SEM			**F**	**p**
**Tail suspension test**							
Freezing time	133.23 ± 8.31	99.23 ± 15.98	116.2 ± 15.02	125.31 ± 12.06	**Treatment**<br>**Genotype**<br>**Treatment X genotype**	0.114<br>0.863<br>0.114	0.737<br>0.357<br>0.114
Freezing episodes	9.23 ± 0.45	7.50 ± 1.047	7.39 ± 0.76	8.31 ± 0.89	**Treatment**<br>**Genotype**<br>**Treatment X genotype**	0.572<br>0.148<br>3.080	0.453<br>0.702<br>0.085
Short freezing episodes	0.85 ± 0.28	0.62 ± 0.25	0.83 ± 0.21	0.92 ± 0.24	**Treatment**<br>**Genotype**<br>**Treatment X genotype**	0.275<br>0.039<br>0.610	0.602<br>0.844<br>0.439
Latency to freeze	38.71 ± 6.11	69.46 ± 14.35	97.83 ± 24.14	75.00 ± 12.79	**Treatment**<br>**Genotype**<br>**Treatment X genotype**	4.189<br>0.063<br>2.877	**0.046**<br>0.803<br>0.096

**Open field test**							
Distance travelled	42.84 ± 5.09	37.11 ± 5.13	35.77 ± 4.96	42.81 ± 6.62	**Treatment**<br>**Genotype**<br>**Treatment X genotype**	0.016<br>0.014<br>1.369	0.901<br>0.906<br>0.247
Distance 5 min	11.07 ± 1.46	10.22 ± 1.39	9.91 ± 1.75	12.76 ± 1.97	**Treatment**<br>**Genotype**<br>**Treatment X genotype**	0.180<br>0.367<br>1.254	0.673<br>0.548<br>0.268
Distance in center 5 min	1.46 ± 0.32	1.02 ± 0.41	1.95 ± 0.40	1.52 ± 0.23	**Treatment**<br>**Genotype**<br>**Treatment X genotype**	0.180<br>0.367<br>1.254	0.673<br>0.548<br>0.673
Corner visits 5 min	7.93 ± 1.82	9.15 ± 1.72	3.57 ± 0.94	9.54 ± 2.24	**Treatment**<br>**Genotype**<br>**Treatment X genotype**	1.329<br>4.356<br>1.893	0.245<br>**0.042**<br>0.175
**Elevated plus maze**							
Open arm frequency	5.14 ± 1.15	5.85 ± 1.23	5.60 ± 1.12	6.92 ± 1.44	**Treatment**<br>**Genotype**<br>**Treatment X genotype**	0.389<br>0.678<br>0.063	0.536<br>0.414<br>0.804
Closed arm frequency	8.72 ± 2.07	12.85 ± 2.64	9.87 ± 2.05	15.58 ± 2.29	**Treatment**<br>**Genotype**<br>**Treatment X genotype**	0.735<br>4.713<br>0.122	0.395<br>**0.035**<br>0.728

Protected head tips	3.50 ± 0.81	3.46 ± 0.60	5.07 ± 1.64	5.33 ± 0.62	**Treatment**	2.533	0.118
					**Genotype**	0.011	0.916
					**Treatment X genotype**	0.020	0.888
Unprotected head tips	0.64 ± 0.29	1.69 ± 1.02	1.53 ± 0.80	0.33 ± 0.19	**Treatment**	0.118	0.733
					**Genotype**	0.012	0.913
					**Treatment X genotype**	2.715	0.106
